# ZYG11B suppresses multiple enteroviruses by triggering viral VP1 degradation

**DOI:** 10.1128/jvi.00030-25

**Published:** 2025-03-26

**Authors:** Li Tian, Zhizhong Mi, Weijing Yang, Jing Chen, Xiulong Wei, Wenyan Zhang, Zhaolong Li

**Affiliations:** 1 Institute of Virology and AIDS Research, the First Hospital of Jilin University 664674 , Changchun, Jilin, China; 2 Department of Infectious Diseases, Infectious Diseases and Pathogen Biology Center, Key Laboratory of Organ Regeneration and Transplantation of The Ministry of Education, The First Hospital of Jilin University 117971 , Changchun, Jilin, China; University of Michigan Medical School, Ann Arbor, Michigan, USA

**Keywords:** ZYG11B, CRL2, EV71, enterovirus, ubiquitination

## Abstract

Enterovirus 71 (EV71) is a major cause of hand, foot, and mouth disease, particularly affecting pediatric populations worldwide. The role of ZYG11B, a CUL2-complex-associated E3 ubiquitin ligase from the Zyg-11 family, in antiviral defense against EV71 remains unclear. To our knowledge, this study is the first to reveal that ZYG11B targets EV71 VP1 for proteasomal degradation via the ubiquitin-proteasome pathway, with CRL2^ZYG11B^ complex activity specifically driving K33-linked ubiquitination. Mass spectrometry and immunoprecipitation analyses confirmed the interaction between ZYG11B and VP1 and identified key domains required for binding both VP1 and CUL2. Comparative analyses showed that VP1 ubiquitination sites are highly conserved across related enteroviruses, including CA6, CA16, and EVD68. Functional assays further demonstrated that ZYG11B restricts these viruses, highlighting its potential as a broad-spectrum antiviral target. These findings establish ZYG11B as a critical effector in host antiviral responses and support its therapeutic potential for managing enterovirus infections.

**IMPORTANCE:**

E3 ubiquitin ligases and deubiquitinases have become important topics of competition between viruses and hosts. Here, we identified CRL2^ZYG11B^ as an E3 ubiquitin ligase complex capable of degrading structural protein VP1 of enteroviruses, making ZYG11B a broad-spectrum antiviral factor. We first proposed the inhibitory effect of ZYG11B on viruses and identified the structural domains of ZYG11B connecting substrates and CUL2, providing new targets for the design of antiviral drugs.

## INTRODUCTION

Enteroviruses, belonging to the Picornaviridae family, pose a significant global health threat because of their wide distribution and ability to cause a broad spectrum of diseases. These RNA viruses, including enterovirus 71 (EV71), coxsackievirus, and poliovirus, are predominantly transmitted via the fecal–oral route. Infections can lead to various clinical outcomes, ranging from mild respiratory or gastrointestinal symptoms to more severe neurological conditions, such as aseptic meningitis, encephalitis, and acute flaccid paralysis (1). In particular, EV71, a single-stranded RNA virus, has emerged as a major pathogen in outbreaks of hand, foot, and mouth disease (HFMD), with severe cases leading to fatal neurological complications, especially in infants and young children, because of its lack of a proofreading mechanism (2). The EV71 genome encodes structural proteins VP1–VP4, which form the viral capsid, and a series of non-structural proteins that drive replication and processing. VP1 is the primary immunogenic component essential for viral entry and immune targeting. VP2 and VP3 contribute to capsid integrity and receptor binding, whereas VP4 stabilizes the viral RNA and aids in its release upon infection. Non-structural proteins (2A–2C and 3A–3D) facilitate RNA synthesis, protein cleavage, and immune evasion, ensuring efficient viral replication and propagation (3).

Targeting the ubiquitin-proteasome system (UPS), a key regulator of protein homeostasis, offers a potential strategy to combat these infections. Ubiquitination, the process of tagging proteins with ubiquitin for proteasomal degradation, plays a complex role during infection (4–6). While enteroviruses can manipulate the UPS to enhance their replication and evade immune detection, host cells utilize this pathway to degrade viral proteins and limit infection (7). Recent evidence underscores the significance of ubiquitin-mediated degradation in restricting viral replication and disease progression, presenting it as a promising antiviral approach (8–10). During EV71 infection, the UPS plays a pivotal role. Studies suggest that EV71 leverages the UPS to enhance its replication and evade host immunity, partly by degrading antiviral host factors (11). Moreover, structural proteins, like VP1, may be regulated through interactions with E3 ubiquitin ligases, affecting their stability within host cells (12).

ZYG11B, a member of the ZYG11 protein family, plays an important role in processes, such as cell cycle regulation and apoptosis. Emerging evidence also suggests its involvement in developmental pathways and diseases (13), highlighting its potential significance in cellular homeostasis and pathological conditions. While ZYG11B has been shown to enhance antiviral immune responses, particularly through pathways, like cGAS-STING (14), its potential role as an E3 ubiquitin ligase in direct antiviral defence has not yet been reported.

In this study, mass spectrometry analysis revealed the potential interact proteins of the structural protein VP1 of EV71. Subsequent experiments showed that ubiquitination promotes the degradation of EV71 VP1. Further investigation identified the specific ubiquitination site on VP1 and the binding region for ZYG11B, confirming that ZYG11B, as a component of the CRL2 complex, mediates the ubiquitin-dependent degradation of VP1. This degradation mechanism has also been observed in other enteroviruses, such as enterovirus D68 (EV-D68), coxsackievirus A6 (CA6), and coxsackievirus A16 (CA16), suggesting that ZYG11B-mediated ubiquitination may represent a broad-spectrum antiviral strategy and provide new avenues for enterovirus therapy.

## RESULTS

### CRL2^ZYG11B^ E3 ligase mediates VP1 degradation

**Fig 1:**
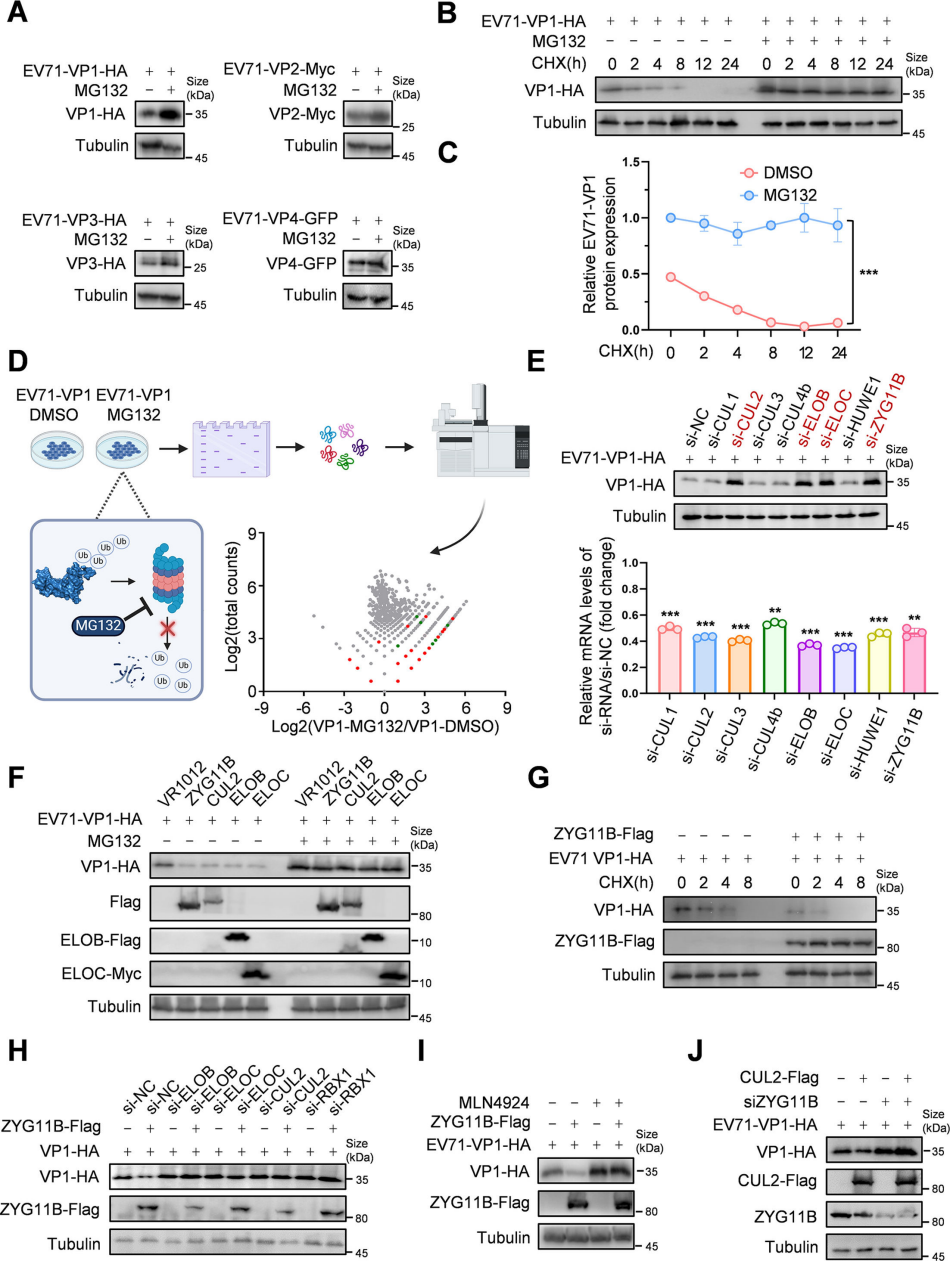
ZYG11B-mediated ubiquitination and proteasomal degradation of EV71-VP1 protein. (A) The proteasomal inhibitor MG132 increases the stability of the EV71-VP1 protein. HEK293T cells were transfected with the indicated plasmids for 36 h and treated with 10 µM MG132 or DMSO for 16 h before being harvested. Protein expression was analyzed by IB. (B and C) MG132 enhances the half-life of the EV71-VP1 protein. Protein expression was analyzed by IB and Image J. (D) Proteins associated with ubiquitin on the EV71-VP1 protein were analyzed using proteomic analysis. The 293T cells were placed in a 10 cm cell culture dish and transfected with EV71-VP1-Flag the next day. The cells were harvested 48 h later and treated with MG132 (10 µM) proteasome inhibitor 16 h before harvesting. The cell lysate was incubated with Flag beads at 4℃ overnight, competed with Flag peptide at 4℃ for 4 h, proteins separated by SDS-PAGE, stained with Coomassie Brilliant Blue, and analyzed by mass spectrometry (MS). E3 proteins are highlighted in red and PSMD proteins in green on the scatter plot. (Created with BioRender.com under agreement no. MQ27R0M62S.) (E) CUL2, ELOB, ELOC, and ZYG11B regulate the stability of VP1. HEK293T cells were transfected with the indicated siRNA for 24 h before transfecting EV71-VP1-HA plasmid. After 48 h, the cells were harvested, and protein expression was analyzed by IB and RT-qPCR. The corresponding protein levels after knockdown are shown in Fig. S1B. (F) ZYG11B, CUL2, ELOB, and ELOC mediate the degradation of EV71-VP1 through the proteasomal pathway. HEK293T cells were transfected with ZYG11B, CUL2, ELOB, or ELOC and EV71-VP1 36 h and treated with MG132 or DMSO for 16 h. (G) Expression of ZYG11B shortens the half-life of the EV71-VP1 protein. HEK293T cells receiving ZYG11B or the negative control VR1012 were transfected with the EV71-VP1 plasmid and then treated with 50 µg/mL of CHX prior to being harvested at the indicated time points. The quantification of protein levels and the half-life analysis using Image J are presented in Fig. S1C. (H) ZYG11B mediates the degradation of the EV71-VP1 protein through the CRL2 complex. HEK293T cells were transfected with EV71-HA and ZYG11B-Flag after being transfected with the indicated siRNA for 24 h. Protein expression was analyzed by IB. The knockdown efficiency of siRNA, assessed by RT-qPCR and IB, is presented in Fig. S1D. (I) The CRL2 inhibitor blocks ZYG11B-mediated degradation of VP1. ZYG11B and EV71-VP1 were co-transfected, and after 36 h, the cells were treated with MLN4924 (10 μM) for 16 h before being harvested. Western blot (IB) analysis was then performed. (J) ZYG11B is required for CUL2 to mediate the degradation of EV71-VP1. The data are representative of three independent experiments, expressed as mean ± standard deviation (*n* = 3). Student *t*-test (unpaired, two-way) was used for comparison between two independent groups, and two-way ANOVA was used for comparison between multiple groups: **P < 0.01; ***P < 0.001.

Previous studies have shown that the proteasome regulates VP2 and VP3 proteins of EV71 (15, 16). To determine whether VP1 or VP4 is similarly regulated, we treated cells with the proteasome inhibitor, MG132, and observed its effects on these proteins (Fig. 1A). The results indicated that VP1 levels were significantly restored upon MG132 treatment. Subsequently, we determined the half-life of VP1 by adding the protein synthesis inhibitor cycloheximide (CHX) and measuring protein levels at various time points. We found that VP1 was almost undetectable at 8 h, but its degradation was significantly inhibited after treatment with MG132 (Fig. 1B and C). To identify E3 ubiquitin ligases that specifically target EV71-VP1, we enriched the protein via co-immunoprecipitation (co-IP) and performed mass spectrometry analysis. Heat map analysis of the mass spectrometry results revealed E3 ubiquitin ligases enriched with VP1 in the MG132 or DMSO treatment groups (Fig. S1A). Volcano plot analysis identified several potential E3 ubiquitin ligases, including CUL1, CUL2, CUL3, CUL4b, ELOB, ELOC, HUWE1, and ZYG11B, with peptide segments enriched more than 10-fold (highlighted as red dots, Fig. 1D). We also observed an enrichment of several proteins associated with the proteasomal pathway (green dots). This confirms the close relationship between the proteasome pathway and VP1. To assess their role in VP1 regulation, we designed siRNAs targeting these candidate proteins and measured EV71-VP1 levels after their knockdown. We found that knockdown of CUL2, ELOB, ELOC, and ZYG11B restored EV71-VP1 levels (Fig. 1E). Knockdown efficiency was measured by RT-qPCR and Western blot (Fig. S1B). In contrast, overexpression of ZYG11B, CUL2, ELOB, or ELOC promoted VP1 degradation, an effect that was abolished after MG132 treatment (Fig. 1F). We further examined the half-life of VP1 in the presence of ZYG11B and found an accelerated degradation rate, significantly shortening its half-life (Fig. 1G and Fig. S1C). Given previous studies suggesting that ZYG11B can act as a substrate-recognizing subunit in the CRL2 E3 ubiquitin ligase complex (17), we knocked down the components of the CRL2 complex—ELOB, ELOC, CUL2, or RBX1—and then transfected ZYG11B. The results showed that in the absence of any of these components, ZYG11B could not promote VP1 degradation (Fig. 1H and Fig. S1D). We further investigated this by adding inhibitors and found that MLN4924, which blocks Cullin neddylation and inactivates CRLs, prevented ZYG11B from promoting EV71-VP1 degradation (Fig. 1I). We also investigated the role of CUL2 in regulating VP1 by knocking down ZYG11B and found that exogenous CUL2 alone was unable to induce VP1 degradation (Fig. 1J). These findings indicate that ZYG11B, CUL2, ELOB, and ELOC regulate VP1 through the formation of the CRL2^ZYG11B^ complex.

### ZYG11B inhibits EV71 replication

**Fig 2:**
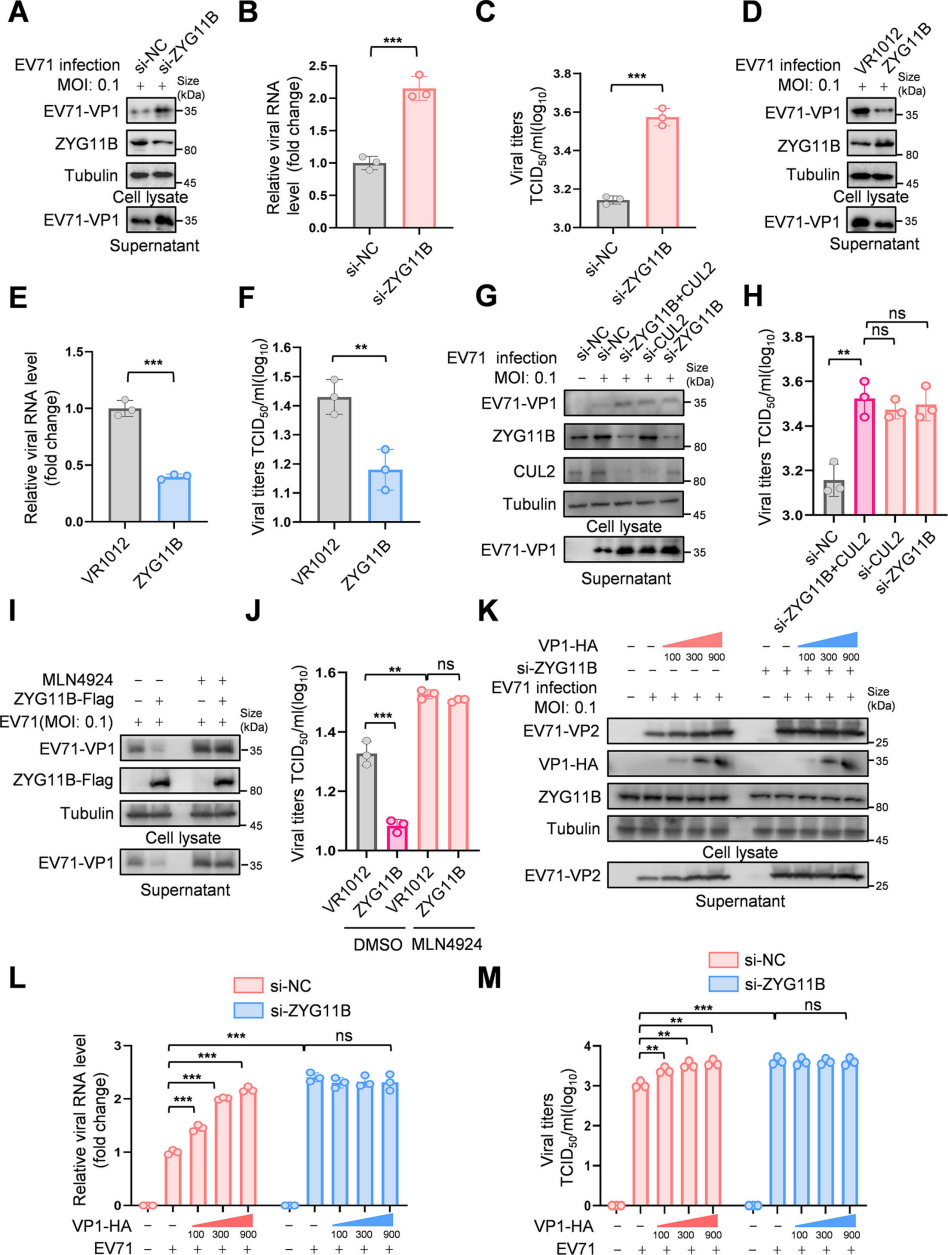
ZYG11B exerts antiviral effects against EV71 through the CRL2 complex. (A-F) ZYG11B inhibits EV71 replication. HEK293T cells were transfected with the indicated plasmids or siRNA and infected with EV71 (MOI: 0.1). After 48 h, cells and supernatant were harvested. EV71-VP1 levels in cells as well as ZYG11B were measured by IB (A and D). The viral RNA level in the supernatant was detected by RT-qPCR (B and E), and the viral titer in the supernatant was detected (C and F). (G and H) ZYG11B and CUL2 cooperatively inhibit EV71 replication. HEK293T cells were transfected with the indicated siRNAs targeting ZYG11B, CUL2, or controls, followed by infection with EV71 (MOI: 0.1) after 24 h. Cells and supernatants were harvested 48 h post-infection. EV71-VP1 and ZYG11B protein levels in cells were analyzed by immunoblotting (IB) (G). The viral titers in the supernatant were measured (H). (I and J) The CRL2 inhibitor MLN4924 blocks the antiviral effect of ZYG11B. After transfecting ZYG11B, cells were infected with the EV71 (MOI: 0.1) 24 h later and treated with MLN4924 (10 µM) or DMSO. The medium was changed 2 h later to include the same concentration of MLN4924, and cells and supernatants were collected after 48 h. EV71-VP1 protein levels in both cells and supernatants were detected by IB (I). The viral titers in the supernatants were measured (J). (K-M) The antiviral effect of ZYG11B depends on the regulation of VP1 protein levels. HEK293T cells were transfected with siRNA targeting ZYG11B or negative control (NC) for 24 h, followed by gradient transfection of EV71-VP1-HA plasmid. Cells were subsequently infected with EV71 (MOI: 0.1). After 48 h, cells and supernatants were harvested. The protein levels of EV71-VP1 in cells and supernatants were analyzed by immunoblotting (IB) (K). Viral RNA levels in the supernatant were quantified using RT-qPCR (L), and viral titers in the supernatant were measured (M). The data are representative of three independent experiments, expressed as mean ± standard deviation (*n* = 3). The Student *t*-test (unpaired, two-way) was used for comparison between two independent groups, and two-way ANOVA was used for comparison between multiple groups: ns; **P < 0.01; ***P < 0.001.

VP1, the capsid protein of EV71, is highly immunogenic and plays a crucial role in viral recognition, adsorption, target cell entry, and viral particle assembly (18). Therefore, we focused on investigating the role of ZYG11B in EV71-VP1 regulation. We knocked down ZYG11B in RD cells, an enterovirus-sensitive cell line (19), and assessed viral replication by measuring VP1 protein levels in both cells and supernatant (Fig. 2A), as well as VP1 RNA levels in the supernatant (Fig. 2B). Additionally, we measured the viral titer in the supernatant (Fig. 2C). Our results showed that knocking down ZYG11B significantly increased viral replication in both supernatant and cells. In contrast, overexpressing ZYG11B in HEK293T cells led to a notable reduction in viral protein levels (Fig. 2D), RNA levels (Fig. 2E), and titers (Fig. 2F), effectively inhibiting viral replication. Since CUL2 regulation of VP1 depends on ZYG11B, we further investigated whether these two proteins influence viral replication together. The results showed that knocking down either CUL2 or ZYG11B independently enhanced viral replication in both the supernatant and cells. When both factors were simultaneously knocked down, the increase in viral replication was comparable to that observed with individual knockdowns (Fig. 2G and H and Fig. S2A). We also found that the CRL inhibitor MLN4924 blocked the antiviral effect of ZYG11B on EV71, indicating that CUL2 and ZYG11B regulate viral replication in an interdependent manner (Fig. 2I and J and Fig. S2B). Additionally, upon infection with EV71, we observed an increase in viral levels with gradient complementation of EV71-VP1. However, when endogenous ZYG11B was knocked down, even with gradient complementation of EV71-VP1, viral levels did not increase (Fig. 2K through M). To further rule out other potential targets of ZYG11B’s antiviral effect, we first examined its impact on viral translation. By assessing the expression of luciferase linked to the 5′ UTR of EV71, we found that ZYG11B did not affect the activity of the 5′ UTR (Fig. S2C). In addition, in the single-round infection model, we assessed the impact of ZYG11B on the protein and RNA levels of VP1 and another key structural protein, VP2. We found that ZYG11B only affected the protein level of VP1, leading to its decrease, while VP2 protein slightly accumulated in cells due to the reduction of VP1. Moreover, the RNA levels of both VP1 and VP2 in the cells remained unaffected, suggesting that ZYG11B’s regulatory effect on the virus depends on its modulation of EV71-VP1 protein levels (Fig. S2D through F).

### ZYG11B acts as a CRL2 adaptor for interacting with VP1

**Fig 3:**
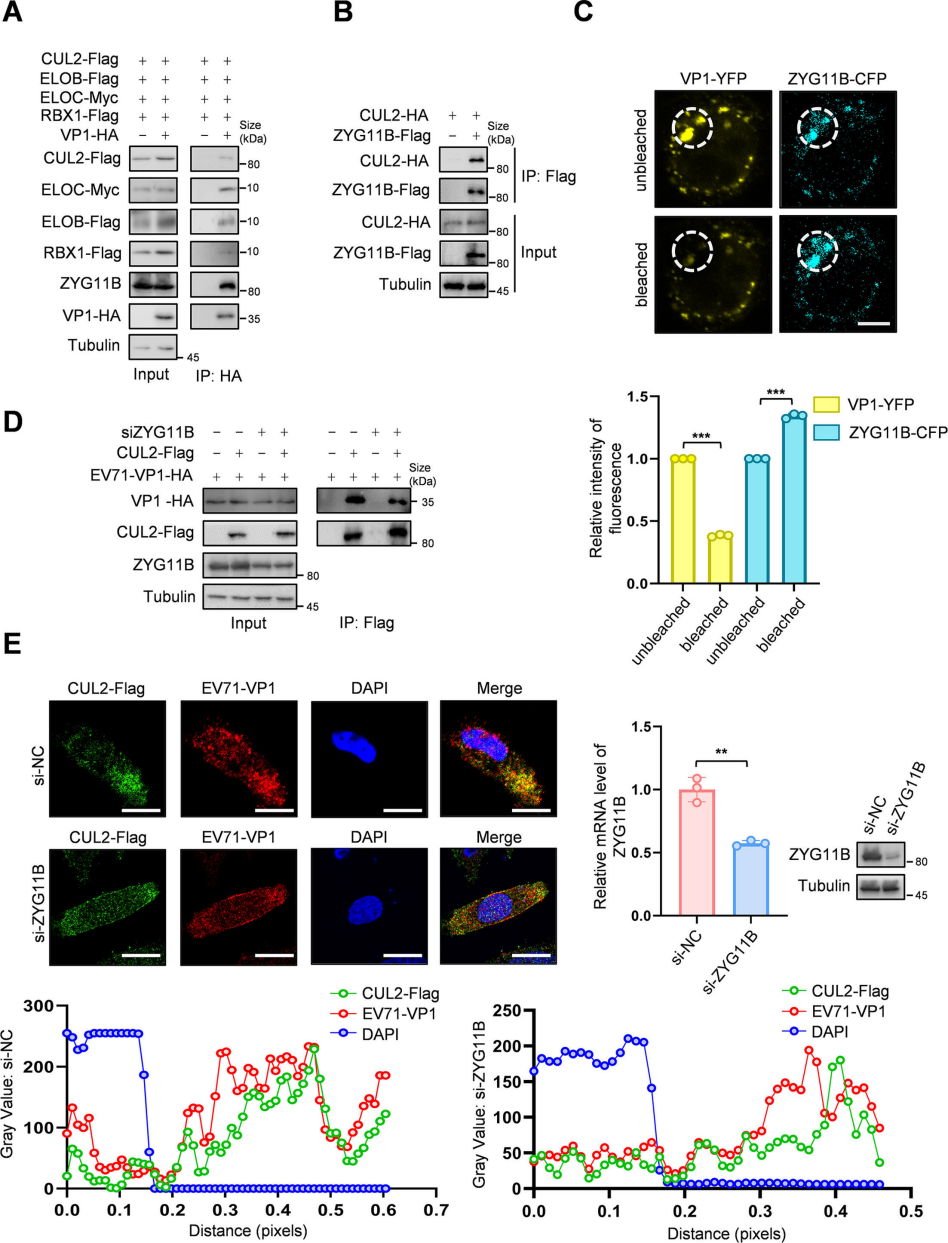
ZYG11B interacts with EV71-VP1 and CUL2. (A) EV71-VP1 interacts with CRL2 complex. HEK293T cells were transfected with the indicated plasmids. After 36 h, MG132 (10 µM) was added, and the cells were treated for 16 h and harvested. EV71-VP1 was enriched by protein-G with HA antibody. Protein expression was analyzed by IB. (B) ZYG11B interacts with CUL2. HEK293T cells were transfected with CUL2-HA, ZYG11B-Flag, and VR1012 for negative control. After 48 h, the cells were harvested. ZYG11B was enriched by protein-G with Flag antibody. Protein was analyzed by IB. (C) FRET experiment demonstrates that VP1 interacts with ZYG11B. Fluorescence was visualized using scanning confocal microscope and analyzed by Image J. The region chosen for photobleaching is marked (white open box). Bars, 5 µm. (D and E) Knockdown of ZYG11B decreased the interaction between EV71-VP1 and CUL2. siRNA of ZYG11B and NC was transfected to HEK293T cells or HELA cells. After 12 h, the cells were transfected with VR1012, CUL2, and EV71-VP1 plasmids for 36 h and then MG132 (10 µM) was added for 12 h. (D) HEK293T cells were harvested, and CUL2 was enriched by protein-G with Flag antibody. Protein was analyzed by IB. (E) For HELA cells, EV71-VP1 and CUL2 were labeled overnight with HA antibody and Flag antibody. Then, they were fluorescently labeled with 488 or 568 fluorescent secondary antibodies. Cell nuclei were stained using DAPI. Representative images are shown. Scale bars, 10 µm. The gray value and ratio of co-localization were quantified by measuring the fluorescence intensities using Image J. The data are representative of three independent experiments, expressed as mean ± standard deviation (*n* = 3). The Student *t*-test (unpaired, two-way) was used for comparison between two independent groups, and two-way ANOVA was used for comparison between multiple groups: **P < 0.01; ***P < 0.001.

The degradation of VP1 via the proteasomal pathway requires the presence of both CUL2 and ZYG11B, suggesting that ZYG11B serves as a crucial component of the CRL2 complex and as a bridge connecting EV71-VP1. To verify this hypothesis, we first discovered through co-IP experiments that the components of the CRL2 complex, including CUL2, ELOB, ELOC, and RBX1, interact with EV71-VP1, including ZYG11B (Fig. 3A). Additionally, ZYG11B was shown to interact with CUL2 (Fig. 3B). To confirm the direct binding between EV71-VP1 and ZYG11B, we conducted a fluorescence resonance transfer (FRET) experiment. When VP1-YFP (yellow) was quenched, ZYG11B-CFP (cyan) fluorescence increased, indicating a direct interaction between EV71-VP1 and ZYG11B (Fig. 3C). Next, we investigated the relationship between EV71-VP1, ZYG11B, and CUL2 by assessing interactions between EV71-VP1 and CUL2 following ZYG11B knockdown. The results showed that reduced ZYG11B expression led to a significant decrease in the interaction between CUL2 and VP1 (Fig. 3D). Furthermore, immunofluorescence co-localization showed a significant overlap between EV71-VP1 (red) and CUL2 (green); however, when ZYG11B was knocked down, the co-localization of VP1 and CUL2 was significantly reduced (Fig. 3E). These findings indicate that the interaction between CRL2 and VP1 depends on ZYG11B.

### CRL2^ZYG11B^ catalyzes the formation of K-33 type ubiquitin chains on EV71-VP1

**Fig 4:**
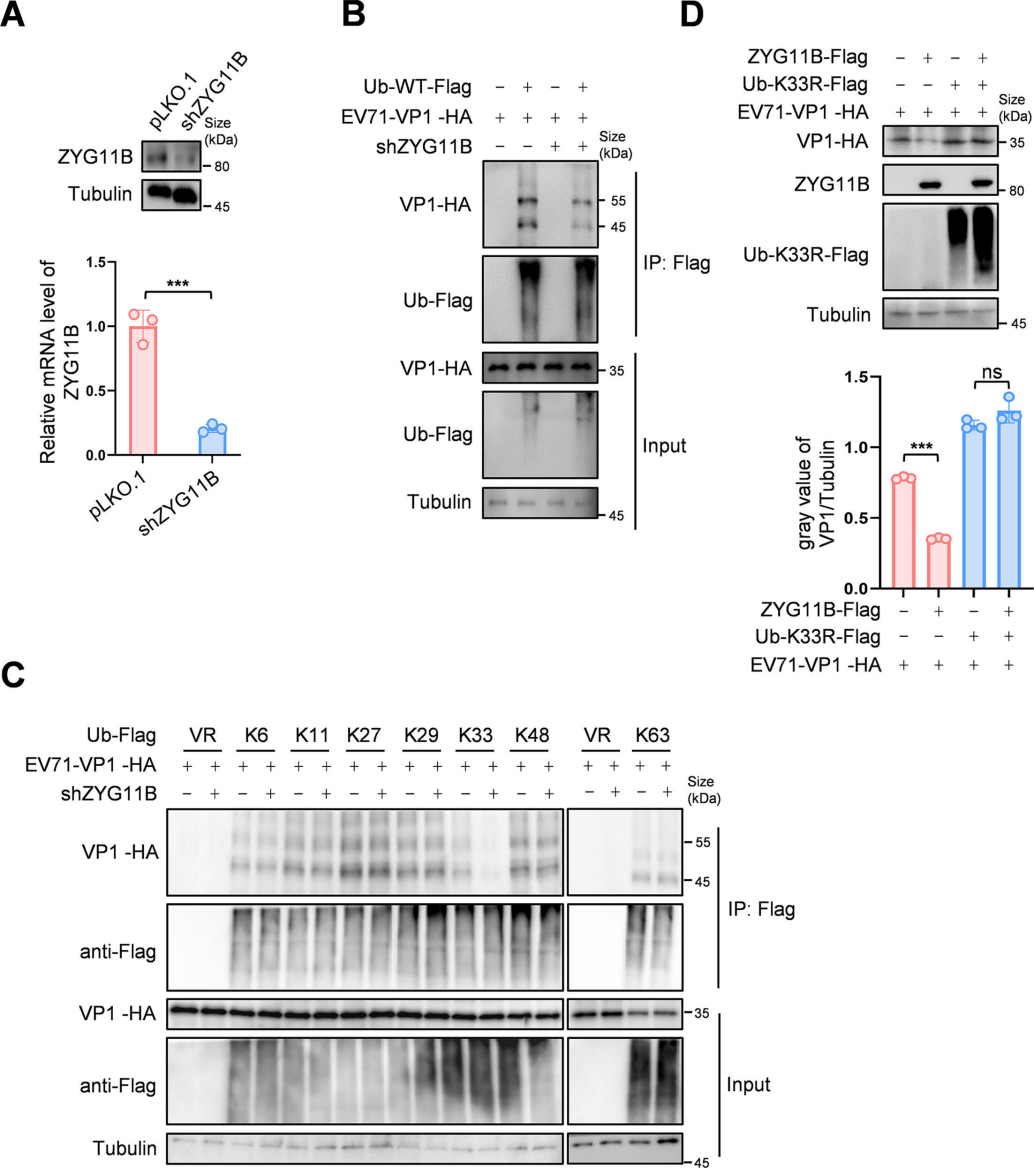
ZYG11B catalyzes the formation of K-33 ubiquitin chains of EV71-VP1. (A) ZYG11B knockdown cell line was constructed, and knockdown efficiency was validated by IB and RT-qPCR. (B) Knocking down ZYG11B affects the ubiquitination level of VP1. Sh-ZYG11B cells were transfected with the indicated plasmids and treated with 10 µM MG132 or DMSO 36 h later for 16 h. After harvesting, Ub was enriched by protein-G with Flag antibody. Protein expression was analyzed by IB. (C) EV71-VP1 was ubiquitinated via K33-linked chains but not K6, K11, K27, K29, K48, or K63. Sh-ZYG11B cells were transfected with EV71-VP1 and Ub-mutants for 36 h, followed by treatment with 10 µM MG132 for 12 h prior to harvest. Cell lysates were immunoprecipitated using protein-G agarose beads conjugated with anti-Flag antibody. Both cell lysates and precipitated samples were analyzed by IB. (D) ZYG11B regulates the degradation of VP1, which is blocked by Ub-K33R. HEK293T cells were transfected with the indicated plasmids for 48 h before being harvested. Protein expression was analyzed by IB and Image J. The data are representative of three independent experiments, expressed as mean ± standard deviation (*n* = 3). Student *t*-test (unpaired, two-way) was used for comparison between two independent groups, and two-way ANOVA was used for comparison between multiple groups: ns; ***P < 0.001.

Although ubiquitin is a small molecule (8.5 kDa), it contains seven lysine residues, enabling the formation of different types of ubiquitin chains (20). These different ubiquitination modifications determine distinct protein functions (21). Using co-IP experiments, we observed both the monoubiquitin and polyubiquitin levels of EV71-VP1. In ZYG11B knockdown cell lines, the overall ubiquitination level of EV71-VP1 was significantly reduced (Fig. 4A and B). To identify the specific type of ubiquitination on EV71-VP1 regulated by ZYG11B, we created mutant Ub plasmids retaining only one lysine residue (-K6, -K11, -K27, -K29, -K33, -K48, and -K63). Upon conducting co-IP experiments in cell lines with ZYG11B knockdown, we found that as ZYG11B levels decreased, K33-type ubiquitination levels also declined, whereas other types of ubiquitination remained unaffected (Fig. 4C). Subsequently, we constructed a Ub mutant, in which the lysine residue at position 33 was mutated to arginine (Ub-K33R), and overexpressed it to assess the ability of ZYG11B to promote VP1 degradation. The results showed that ZYG11B could not promote VP1 degradation in the presence of Ub-K33R (Fig. 4D). These findings confirm that K33-type ubiquitination is essential for ZYG11B-mediated regulation of VP1 degradation.

### Mass spectrometry analysis reveals the ubiquitination site of EV71-VP1

**Fig 5:**
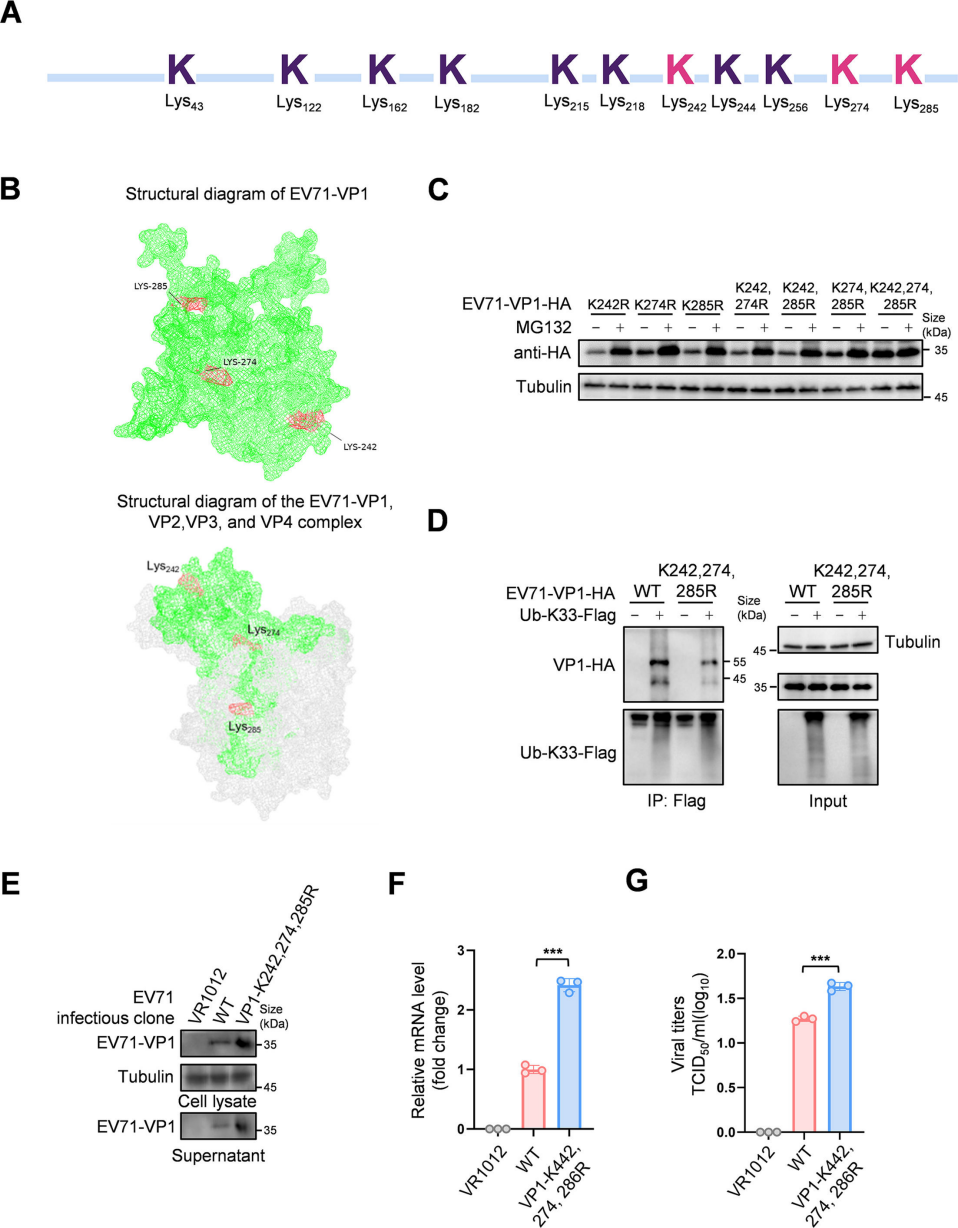
Lysines located at positions 242, 274, and 285 are ubiquitination sites of EV71-VP1. (A) Schematic diagram of lysine distribution in EV71-VP1. The lysine residue highlighted in red is the ubiquitination site obtained by mass spectrometry. (B) Structural analysis of EV71-VP1 protein or the VP1, VP2, VP3, and VP4 complexes to determine the location of ubiquitination sites. The protein structure was obtained from the Protein Data Bank (PDB) website and analyzed using the PyMOL software. (C) All lysine residues located at positions 242, 274, and 285 are mutated to arginine to prevent the degradation of EV71-VP1. Lysine single-site mutations at positions 242, 274, or 285, double-site mutations, and triple-site mutations were obtained through mutation. Corresponding plasmids were transfected into 293T cells. After 36 h, MG132 (10 µM) or DMSO was added for 12 h, and cells were harvested after drug treatment. Protein levels were analyzed using IB and HA antibody. (D) EV71-VP1 shows a significantly decreased K33 type ubiquitination level after mutation of all three lysine residues 244, 274, and 285 to arginine. Ub-K33-Flag and EV71-VP1 wild type or mutant were co-transferred. After 36 h, MG132 treatment was added for 12 h; cells were harvested 48 h after transfection and co-incubated with protein-G containing Flag antibody. EV71-VP1 ubiquitination was analyzed by IB. (E-G) VP1 ubiquitination site mutation promotes replication of infectious clones. The full-length genome of EV71 was ligated onto the VR1012 vector to obtain an infectious clone. Lysine at positions 242, 274, and 285 of the VP1 protein was mutated to arginine, and the infectious clones were transfected into 293T cells. The protein level of the virus VP1 was detected by IB (E), the RNA level in the supernatant was detected by RT-qPCR (F), and the viral titer was measured (G). The data are representative of three independent experiments, expressed as mean ± standard deviation (*n* = 3). Student *t*-test (unpaired, two-way) was used for comparison between two independent groups: ***P < 0.001.

Traditional ubiquitination modifications typically occur on lysine residues (22). EV71-VP1 contains 11 lysine residues. To identify the lysine residues that can be ubiquitinated, we enriched EV71-VP1 through co-IP experiments and performed mass spectrometry analysis. The results identified three sites, Lys242, Lys274, and Lys285, which were screened for ubiquitination modifications (Fig. 5A and Fig. S3A). Structural analysis of EV71-VP1 indicates that these three lysine residues are located on the same side. Upon analyzing the complex formation between VP1, VP2, VP3, and VP4, we found that the positions of these three residues are exposed and situated on the same side, suggesting that they may act synergistically (Fig. 5B). Therefore, we constructed single-, double-, and triple-point mutations. We found that the degradation of EV71-VP1 was completely inhibited only when all three sites were simultaneously mutated (Fig. 5C). Further analysis of K33-type ubiquitination revealed that this modification was almost undetectable when all three lysine residues were simultaneously mutated, confirming that Lys242, Lys274, and Lys285 serve as the K33 ubiquitination sites on VP1 regulated by ZYG11B (Fig. 5D). To underscore the importance of these ubiquitination sites, we constructed an infectious clone of EV71 with lysine-to-arginine mutations at these positions. Upon transfecting these clones, we measured protein and RNA levels in both the supernatant and cells and assessed viral titers (Fig. 5E through G). The results showed that viral replication was significantly enhanced when the ubiquitination sites were mutated.

### The N-terminal 1–58 amino acids of ZYG11B are essential for VP1 interaction

**Fig 6:**
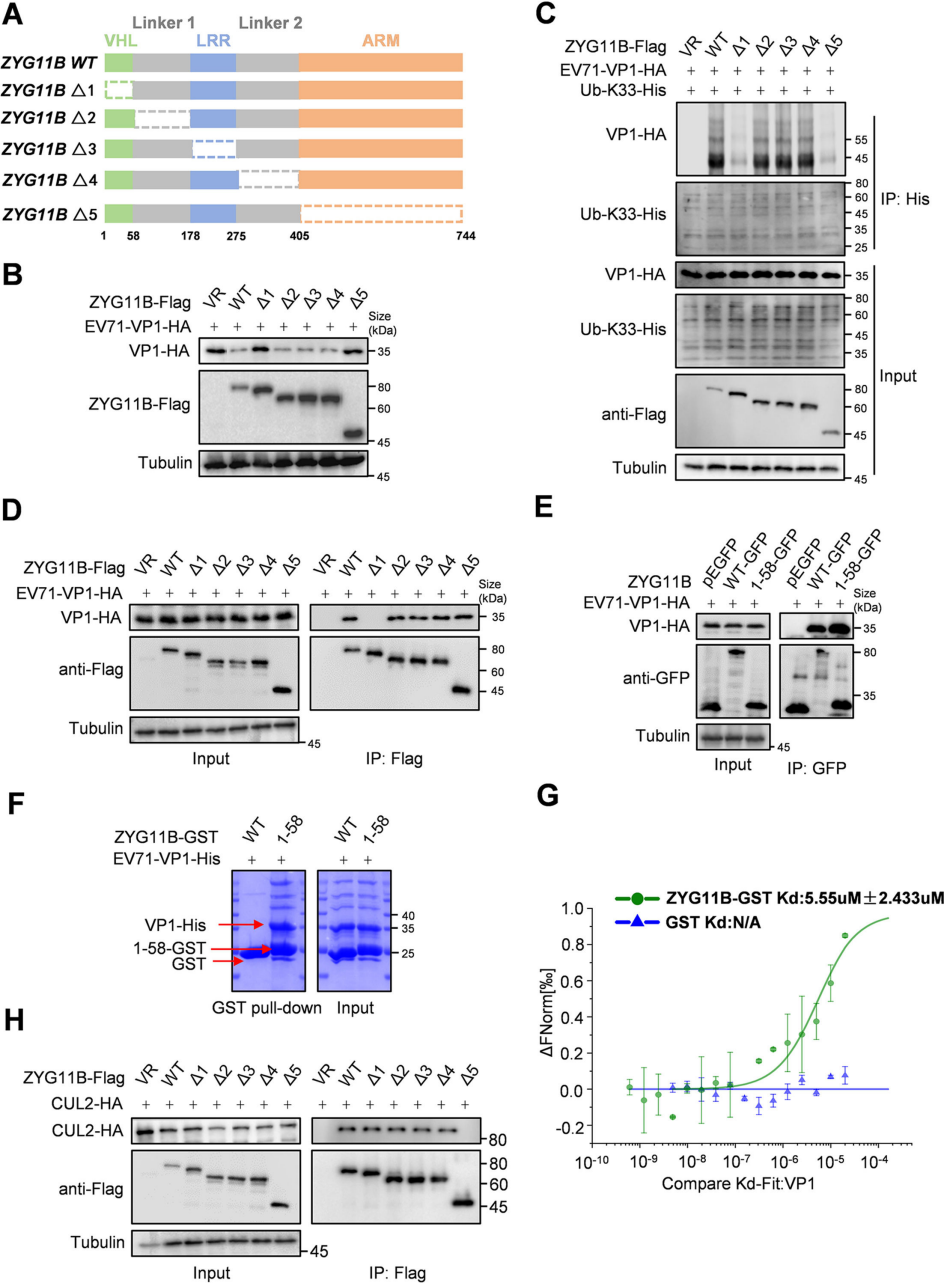
The interaction area between ZYG11B and EV71-VP1 or CUL2. (A) The pattern diagram of the truncated ZYG11B used in this article. (B) ZYG11B truncated forms Δ1 and Δ5 are unable to degrade EV71-VP1. ZYG11B-truncated mutants and EV71-VP1 were transfected in 293T cells, cells were harvested after 48 h, and EV71-VP1 protein levels were detected using IB with HA antibody. (C) The ZYG11B-truncated forms Δ1 and Δ5 cannot increase the level of EV71-VP1 K33 ubiquitination. ZYG11B or the truncated mutant was co-transfected with Ub-K33-His and EV71-VP1-HA in 293T cells. After 36 h, MG132 (10 µM) was added and treated for 16 h before the cells were harvested. Protein enrichment was performed using protein-G with His antibodies. Protein levels were analyzed through IB analysis. (D) ZYG11B-truncated mutant Δ1 does not interact with EV71-VP1. ZYG11B or its mutants and EV71-VP1 were co-transfected into 293T cells. After 36 h, MG132 (10 µM) was added, and the cells were treated for 16 h. After drug treatment, the cells were harvested, and the cell lysate was incubated with protein-G containing Flag antibody to enrich ZYG11B or its truncated mutants. The protein interaction was detected by IB using HA antibody and Flag antibody. (E) The short peptide 1–58 of ZYG11B can interact with EV71-VP1. ZYG11B 1–58 short peptide was constructed onto pEGFP-C1 vector by enzymatic ligation. EV71-VP1 and ZYG11B 1–58 short peptide or ZYG11B-WT were co-transfected in 293T cells. After 36 h, MG132 (10 µM) was added for treatment, and cells were harvested 48 h after transfection. Cell lysate was incubated with protein-G containing GFP antibody, and its interaction was detected through IB analysis. (F) ZYG11B 1–58 short peptide interacts with EV71-VP1 *in vitro*. Using a prokaryotic protein expression system *in vitro*, ZYG11B 1–58 short peptide with GST tag and EV71 VP1 protein with His tag were purified. EV71-VP1-His with GST protein or ZYG11B 1–58-GST protein was incubated *in vitro* for 30 min, and then incubated with GST purification resin at 4℃ for 4 h. After centrifugation at 800 g for 1 min, the supernatant was removed and washed 8–10 times with 1 mM GSH buffer. The proteins were separated by SDS-PAGE and stained by Coomassie Brilliant Blue staining. (G) The interaction between ZYG11B 1–58 and EV71-VP1 *in vitro* was verified using MicroScale thermophoresis (MST). First, the purified protein EV71-VP1 *in vitro* was fluorescently labeled. GST or ZYG11B 1–58-GST was diluted in a twofold concentration gradient and mixed with an equal volume of EV71-VP1. After being aspirated into a capillary tube, it was detected and analyzed using the instrument MicroScale thermophoresis monolith NT.115. (H) ZYG11B-truncated mutant Δ5 does not interact with CUL2. CUL2-HA and ZYG11B or their truncated mutants were co-transfected into 293T cells, and the cells were harvested 48 h later. MG132 (10 µM) was added 12 h before harvesting the cells. Cell lysate was incubated with protein-G containing Flag antibody overnight and tested through IB analysis.

Given that ZYG11B acts as a bridge protein between CUL2 and EV71-VP1, we aimed to identify the specific domains responsible for its interaction with both proteins. ZYG11B contains VHL boxes, four variant leucine-rich repeat (LRR) motifs, and an armadillo-like helical domain (ARM). Based on the existing literature, ZYG11B truncation mutants were generated (Fig. 6A) (14) and tested for their ability to regulate EV71-VP1 degradation and K33-type ubiquitination. We found that deleting amino acid sequences 1–58 (VHL) and 405–744 (ARM) rendered ZYG11B incapable of promoting EV71-VP1 degradation (Fig. 6B) and enhancing K33-type ubiquitination of VP1, highlighting the critical functions of these two domains (Fig. 6C). To confirm whether the absence of the region spanning 1–58 aa or 405–744 aa affects the interaction between ZYG11B and VP1, we performed co-IP experiments. The results indicated that the region spanning 1–58 aa plays a key role in binding VP1 (Fig. 6D). To further validate this, we constructed a 1–58 aa short peptide fused to the pEGFP vector for co-IP experiments. The results showed that the isolated 1–58 aa peptide can effectively bind to EV71-VP1 (Fig. 6E). Immunofluorescence localization analysis revealed that the degree of co-localization between ZYG11B and VP1 was significantly reduced when 1–58 aa were deleted. In contrast, the co-localization of the isolated 1–58 peptide with VP1 was comparable to that observed between ZYG11B wild type and VP1 (Fig. S4A). To confirm their direct interaction, we purified the 1–58 aa peptide and EV71-VP1 using a prokaryotic expression system and validated their interaction *in vitro* through pull-down assays (Fig. 6F) and microscale thermophoresis experiments (Fig. 6G). To further investigate the role of 405–744 aa, we examined the interaction between the truncated forms of ZYG11B and CUL2 using co-IP assays. The results showed that the absence of the region spanning 405–744 aa prevents ZYG11B from binding to CUL2, thereby disrupting its regulation of EV71-VP1 degradation (Fig. 6H). The co-localization of ZYG11B and CUL2 further confirmed the critical role of the ARM region in mediating the interaction between ZYG11B and CUL2 (Fig. S4B). To better illustrate the interactions among ZYG11B, VP1, and CUL2, we performed molecular docking based on the protein structures reported in the Protein Database (PDB, www.rscb.org). The docking results indicated that the 1–58 aa region is essential for its interaction with VP1, while the 405–744 aa region is crucial for binding to CUL2. Additionally, docking analysis identified the 198–207 aa of VP1 as the key interface for interacting with ZYG11B (Fig. S4C).

### ZYG11B triggers the degradation of multiple VP1 molecules

**Fig 7:**
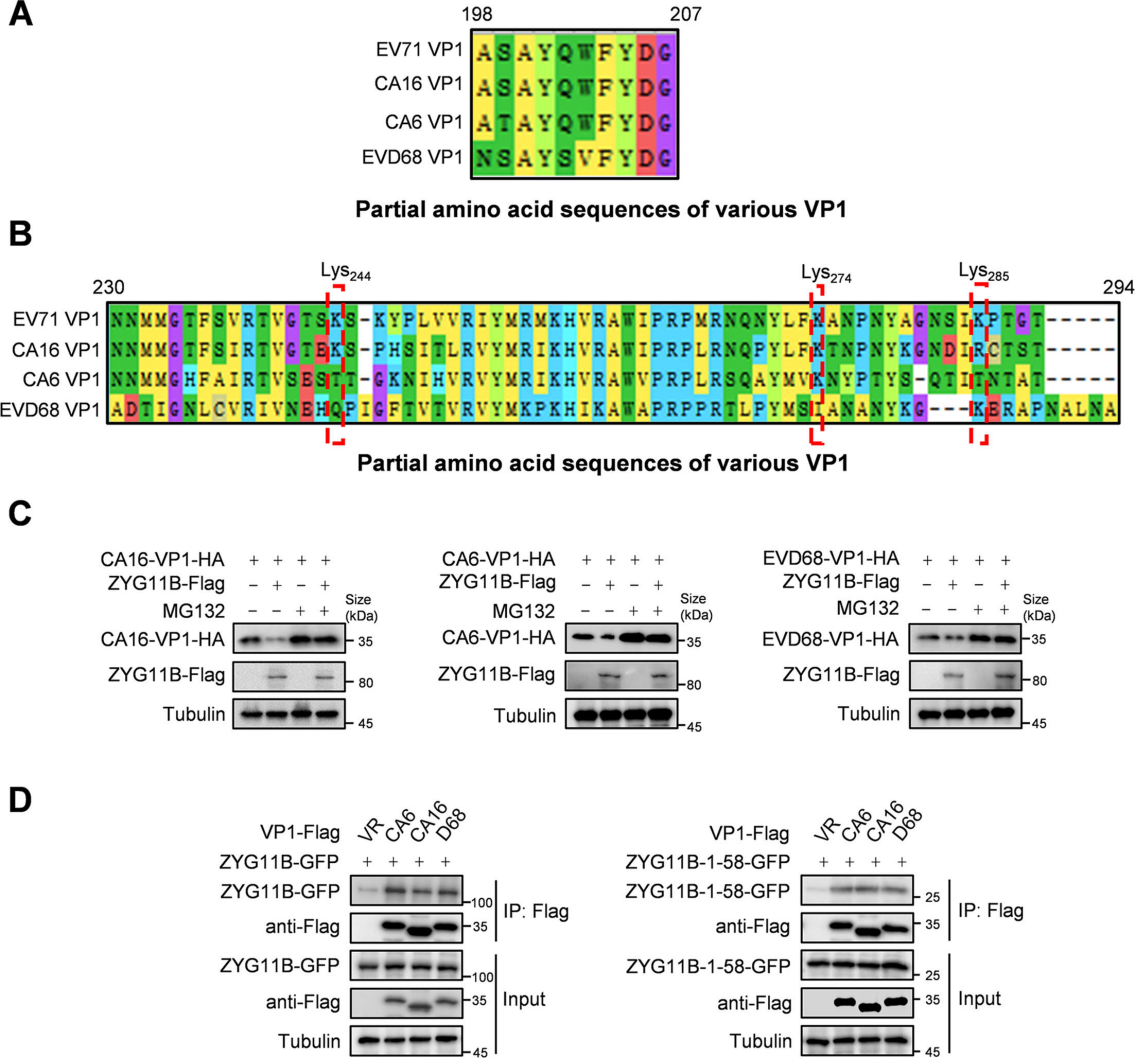
ZYG11B has a degradation effect on VP1 of EV71, EVD68, CA6, or CA16. (A and B) The regions in the VP1 sequences of EV71, EVD68, CA6, and CA16 that interact with ZYG11B are conserved, and the ubiquitination sites are also relatively conserved. VP1 amino acid sequences of EV71, EVD68, CA16, and CA6 were compared using Molecular Evolutionary Genetics Analysis software. (C) ZYG11B degrades the VP1 proteins of EVD68, CA6, or CA16, and this degradation is blocked by MG132 proteasome inhibitors. ZYG11B and VP1 or single transfect VP1 was co-transfected to 293T cells, and the cells were harvested after 48 h. Before harvesting, they were treated with MG132 (10 µM) or DMSO for 12 h. The protein levels of different enterovirus VP1 were detected through IB analysis. (D) The ZYG11B or its 1–58 truncated mutants also interact with other enterovirus VP1. In 293T cells, ZYG11B or ZYG11B 1–58 truncated mutants were transfected singly or co-transfected with different enterovirus VP1. After 36 h, MG132 (10 µM) was added, and the cells were treated for 16 h and harvested. ZYG11B or 1–58 truncated mutants were enriched by protein-G with GFP antibody. Protein analysis used IB with GFP antibody and HA antibody.

EV71, CA6, and CA16 are major causative agents of hand, foot, and mouth diseases (23), whereas EVD68 is associated with severe respiratory diseases (24). Despite ongoing research into antiviral drugs for these viruses, the challenges posed by viral evolution remain unresolved, underscoring the need for broad-spectrum antiviral approaches (25). The amino acid sequences of EV71, EVD68, CA6, and CA16 were retrieved from the National Center for Biotechnological Information (NCBI) database (www.ncbi.nlm.nih.gov). While EV71, CA6, and CA16 share high sequence similarity, EVD68 is less similar. Surprisingly, the VP1 peptide that interacts with ZYG11B is highly conserved across all four virus species (Fig. 7A). Moreover, the ubiquitinated sites Lys244, Lys274, and Lys285 of EV71-VP1 are conserved across other virus species, suggesting that the VP1 of these viruses may also be regulated by the CRL2^ZYG11B^ E3 ligase (Fig. 7B). To validate this hypothesis, we co-transfected a ZYG11B expression plasmid with VP1 from different viruses into HEK293T cells and found that the overexpression of ZYG11B significantly reduced the VP1 protein levels of EVD68, CA6, and CA16. However, this reduction was reversed after treatment with MG132, confirming that ZYG11B promotes the degradation of VP1 from these viruses through the proteasome pathway (Fig. 7C). Additionally, co-IP experiments validated the interaction between the VP1 proteins of EVD68, CA6, or CA16 and the 1–58 aa peptide of ZYG11B (Fig. 7D).

### ZYG11B inhibits multiple enteroviruses

**Fig 8:**
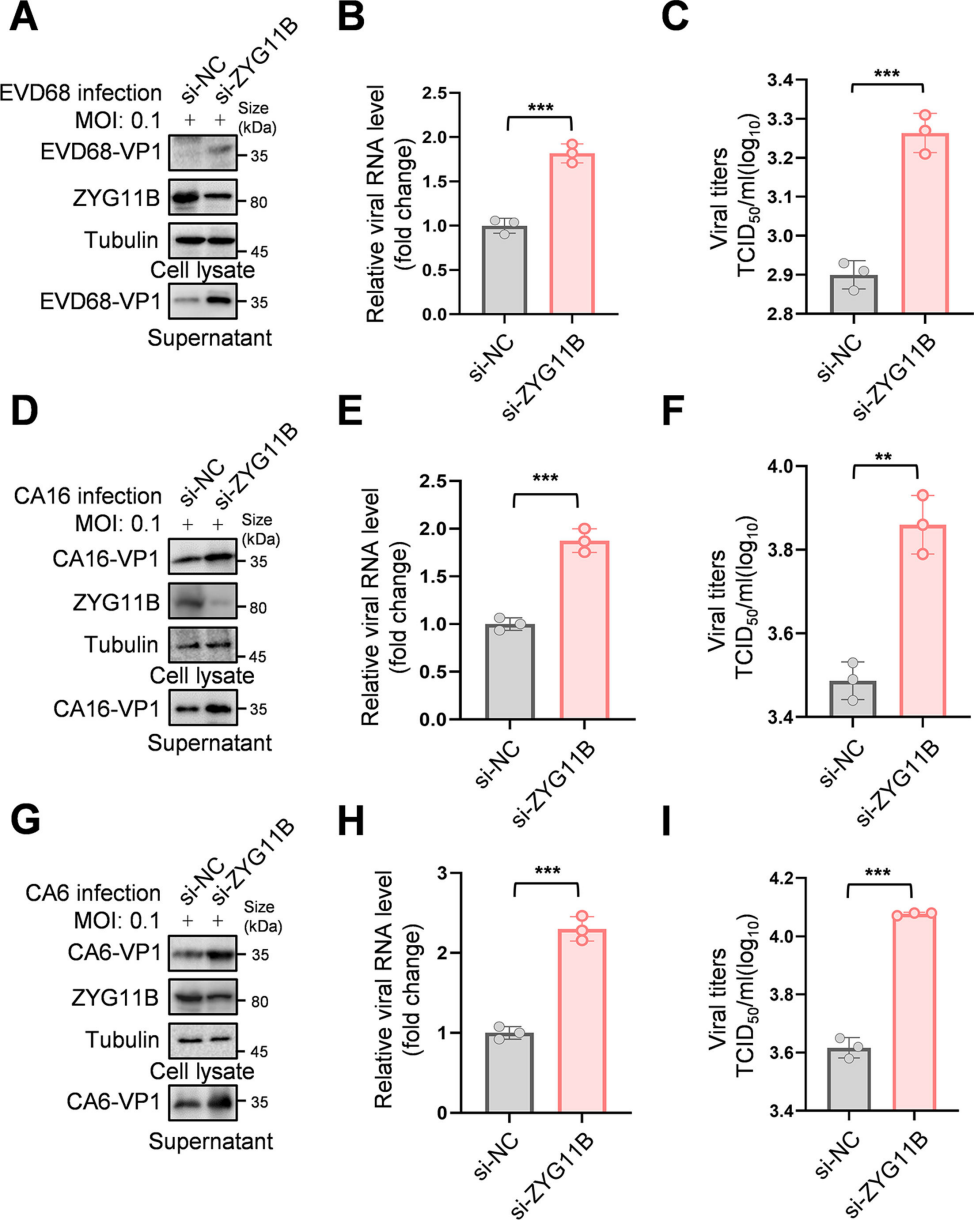
ZYG11B exerts an antiviral effect in EVD68, CA16, and CA6. (A-I) ZYG11B has a broad-spectrum antiviral effect in RD cells. RD cells were transfected with siRNA targeting ZYG11B or NC (a random sequence without targeting) and infected with EVD68 (A-C), CA16 (D-F), and CA6 (G-I) (MOI 0.1) 24 h later. The supernatant was changed to clean culture medium 8 h after infection. Cells and the supernatant were harvested 48 h later. EV71-VP1 levels in cells as well as ZYG11B were measured by IB. The viral RNA level in the supernatant was detected by RT-qPCR, and the viral titer in the supernatant was detected. The data are representative of three independent experiments, expressed as mean ± standard deviation (*n* = 3). Student *t*-test (unpaired, two-way) was used for comparison between two independent groups: **P < 0.01; ***P < 0.001.

To further explore the broad-spectrum antiviral potential of ZYG11B, RD cells were transfected with siRNA targeting ZYG11B. Knockdown of ZYG11B RNA and protein led to a significant increase in VP1 protein levels in both cells and supernatants for EVD68, CA6, and CA16 (Fig. 8A, D and G), as well as a corresponding increase in VP1 RNA levels in the supernatant (Fig. 8B, E and H). Consistent with these findings, viral titers also increased significantly (Fig. 8C, F and I). These results indicate that the antiviral effect of ZYG11B extends to EVD68, CA6, and CA16, highlighting its broad-spectrum antiviral effect. Similar results were obtained in HEK293T cells (Fig. S5A through I). In contrast, the overexpression of ZYG11B reduced the structural protein VP1 levels in EVD68, CA6, and CA16 (Fig. S5J, M and P), decreased VP1 RNA levels in the supernatant (Fig. S5K, N and Q), and lowered viral titers (Fig. S5L, O and R), further confirming the broad-spectrum antiviral activity of ZYG11B.

Enteroviruses are among the most common viruses infecting humans worldwide, with over 100 identified types (26). The high mutation rate of EVs poses a significant challenge to antiviral drug development (27). Recently, several new strains have emerged, with EV71 being particularly prevalent in recent years. The major circulating subtypes include B4, B5, C1, C2, C4, and C5 for EV71; A2, B1, and B3 for EVD68; B, D3a, and D3b for CA6; B1a, B1b, B1c, and B2 for CA16 (19, 28–30); coxsackievirus B (CVB)1–5, coxsackievirus A10 (CA10) and poliovirus (PV) (31–39). We retrieved the amino acid sequences of the structural protein VP1 for various subtypes from the NCBI database and compared them. The analysis revealed that the VP1 peptide interacting with ZYG11B is relatively conserved, with at least one of the ubiquitination sites Lys244, Lys274, or Lys285 present in each subtype (Fig. S6A and B). These findings highlight the broad-spectrum nature of ZYG11B, reinforcing its importance as a promising target for antiviral treatment.

### Enterovirus modulates host antiviral response by downregulating ELOB RNA in the CRL2^ZYG11B^ complex

The interaction between host antiviral mechanisms and enterovirus replication represents an ongoing arms race. To investigate whether enteroviruses suppress ZYG11B (40, 41), we infected RD cells with EV71 and assessed ZYG11B RNA and protein levels at various time points. Our results showed that EV71 does not downregulate ZYG11B levels (Fig. S7A). Similarly, when RD cells were infected with other viruses, including EVD68, CA6, and CA16, ZYG11B RNA and protein levels remained unaffected after 24 h (Fig. S7B). To understand why the antiviral effect of ZYG11B cannot be fully exerted, we focused on the components of the CRL2^ZYG11B^ complex. As ZYG11B functions as an E3 ubiquitin ligase by forming a complex with CUL2, ELOB, ELOC, and RBX1, we analyzed the RNA levels of these factors at various time points following EV71 infection. Interestingly, while the RNA level of CUL2 was upregulated, ELOB RNA levels were significantly downregulated, impairing the functionality of the complex (Fig. S7C). Similar results were also observed after infection with EVD68, CA6, and CA16, indicating a shared mechanism among these viruses (Fig. S7D).

## DISCUSSION

**Fig 9:**
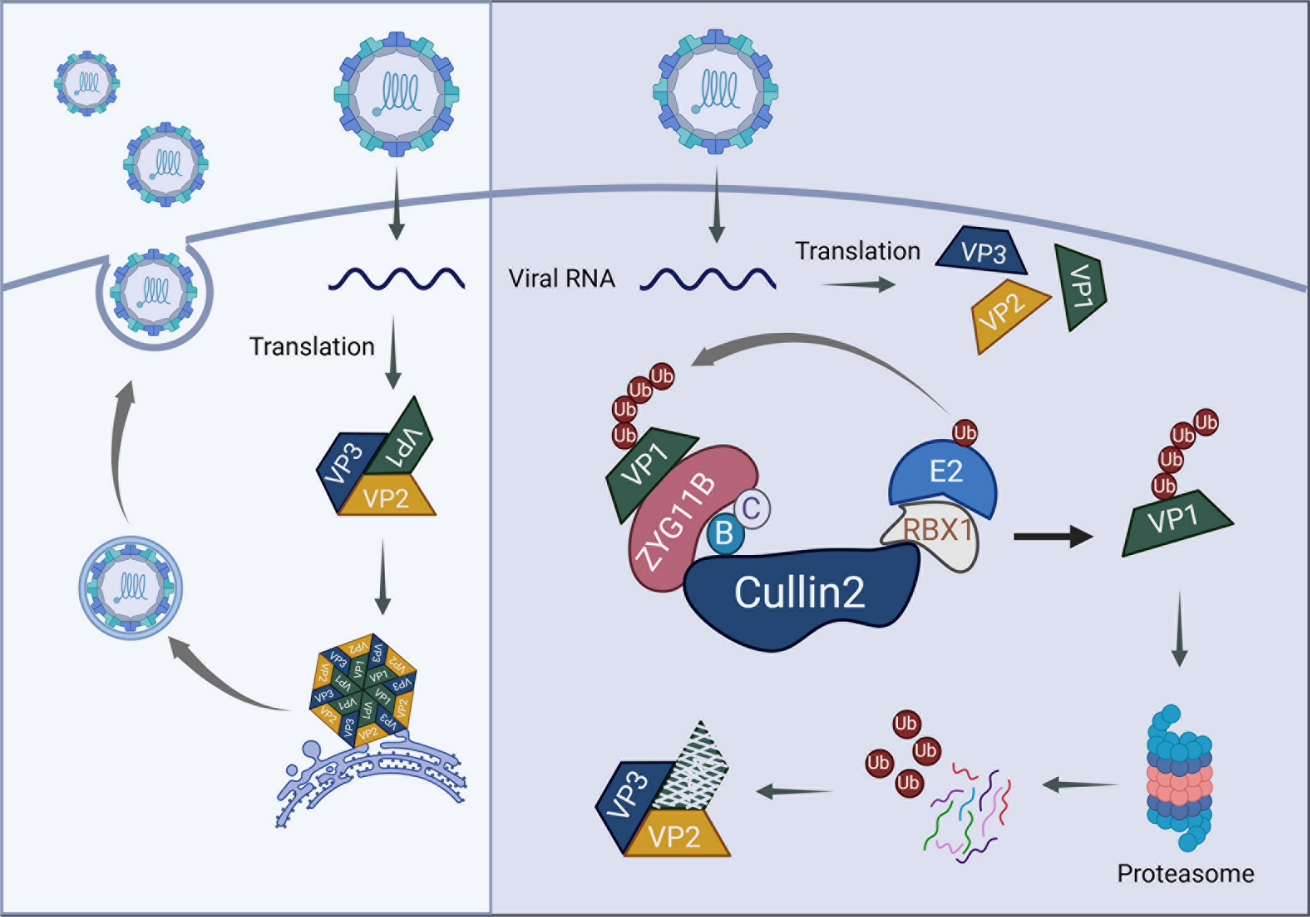
Schematic representation of the CRL2^ZYG111B^ complex mediating EV71-VP1 degradation through the proteasome pathway, resulting in a failure to assemble the virus. (Created with BioRender.com under agreement no. PT27R0MDZP.)

As one of the structural proteins of EV71, VP1 is a key target for vaccine development owing to its high conservation and numerous epitopes (42, 43). Additionally, VP1 plays a crucial role in viral host cell entry and assembly. Mutations in the VP1 gene can impair the virus’s ability to bind to its receptors, thereby reducing its virulence. Furthermore, VP1 allows the virus to evade the host immune response (44), highlighting its significance. Although several drugs and vaccines targeting VP1 and vaccines have been studied, including PTC-209HBr, which inhibits VP1 from binding to the cell receptor hSCARB2 in the early stages of infection (45). The synthetic peptide SP40 significantly inhibits EV71 infection by blocking viral attachment to host cells, with its antiviral activity relying on positively charged amino acids, such as arginine and lysine (46). Moreover, the hydrophobic pocket of VP1 serves as a critical target for drug development (47). However, regarding the relationship between host restriction factors and VP1, it has only been found that the SAMHD1 binding to VP1 disrupts the interaction between VP1 and VP2, thereby impairing viral assembly (48). In response, the virus has evolved a countermeasure by promoting TRIM21 expression, which leads to the degradation of SAMHD1 (49). This interplay suggests the potential involvement of additional, yet unidentified host factors in regulating viral replication. Our study identified that the CRL2^ZYG11B^ complex, an E3 ubiquitin ligase, is a key regulator that ubiquitinates VP1, inducing its proteasome-mediated degradation.(Fig. 9) ZYG11B, the substrate-targeting component of this complex, exhibited robust antiviral activity, as validated by *in vitro* assays. These findings position ZYG11B as a promising target for antiviral drug development, expanding our understanding of host–virus interactions and opening new avenues for therapeutic interventions.

Ubiquitination plays a crucial role in the interplay between viruses and their hosts, impacting pathogens, such as HIV-1, SARS-CoV-2, Zika virus, and dengue virus. Enteroviruses are no exception (50). Research indicates that the 3D protein of EV71 undergoes both SUMOylation and K63-type ubiquitination, enhancing its stability and promoting viral replication (51). Additionally, the 2A protein of EV71 is ubiquitinated by SPOP, a component of the CRL2 E3 ubiquitin ligase complex, leading to its degradation (52). ZYG11B has been identified as one of the constituent components of the CRL2 complex for substrate recognition, and the CRL2^ZYG11B^ complex has been implicated in assisting SARS-CoV-2 infection by causing ciliary dysfunction (53), degrading cyclin B1, and facilitating mitotic slippage (54). In addition to forming the E3 complex, ZYG11B activates interferon production (14). However, the antiviral role of the CRL2^ZYG11B^ complex has not been previously reported. To our knowledge, this study is the first to reveal that CRL2^ZYG11B^ degrades VP1 to inhibit multiple enteroviruses, identifying ZYG11B as a host-restrictive factor.

Cullin-2, a member of the Cullin family, forms an E3 ubiquitin ligase complex with ELOB, ELOC, RBX1, and substrate-specific adaptor proteins. The best-characterized substrate recognition receptor for CRL2 is VHL (55). Notably, ZYG11B contains a VHL domain, and recent findings highlight its interaction with the CUL2–ELOB–ELOC complex (31). Moreover, ZYG11B is known to target substrates with glycine-specific N degrons for proteasomal degradation (17, 56, 57). In our study, despite the presence of a glycine residue at the N-terminus of EV71-VP1, our results suggest that the 1–58 aa region of ZYG11B interacts with the 198–207 aa region of VP1, indicating that ZYG11B may not rely solely on N-terminal recognition for substrate targeting. Consistent with this, a previous study reported that ZYG11B deviates from the N-terminal recognition rule when degrading cyclin B1 (56). Therefore, determining whether EV71-VP1 is recognized by ZYG11B through traditional binding methods or alternative mechanisms will be the focus of our future research. In this study, we constructed ZYG11B mutants to identify their substrate-binding sites and found that ZYG11B1 can bind to VP1 without disrupting its interaction with CUL2, indicating a binding mechanism distinct from previously reported conventional binding sites. However, to ensure the authenticity of our results, we validated the data using *in vitro* pull-down and microscale thermophoresis assays. We hypothesize that ZYG11B may employ non-classical binding modes owing to the diverse nature of its recognized substrates.

Enteroviruses belong to the Picornaviridae family, comprising over 280 viruses that infect humans (58). These RNA viruses, which have circulated across various time periods and regions, are characterized by high mutation rates (59), highlighting the importance of identifying new broad-spectrum antiviral targets. The VP1 protein, a key component of picornavirus capsid proteins, is located on the virus’s outer surface, offering excellent surface accessibility and immunogenicity (60). In this study, ZYG11B demonstrated antiviral activity against EV71, CA6, CA16, and EVD68, with amino acid sequence analyses showing that the interaction regions and ubiquitination sites are highly conserved among these strains. Given the global outbreaks of enterovirus infections, particularly in Russia, we further examined additional enteroviruses, including CVB, CA10, and PV. Remarkably, the interaction regions and ubiquitination sites in these viruses were also found to be relatively conserved, reinforcing the potential of ZYG11B as a broad-spectrum antiviral effector targeting viral protein features, offering valuable insights for the development of broad-spectrum antiviral drugs and immunotherapies. Our findings revealed that ELOB downregulation following infection with various viral strains impairs the antiviral activity of CRL2^ZYG11B^, highlighting virus–host interactions that may disrupt immune responses. Further exploration into the viral factors affecting ELOB and the host pathways regulating its stability could pave the way for developing strategies to counteract viral interference and enhance broad-spectrum antiviral defenses. In summary, this study sheds light on the potential pathogenic mechanisms of host–virus protein interactions, offering new perspectives for antiviral drug development.

## MATERIALS AND METHODS

### Plasmid construction

Full-length ZYG11B was amplified by PCR using the pFLAG-ZYG11B plasmid, obtained from Addgene (plasmid #110550) and cloned into the VR1012 vector between the SalI and NotI restriction sites. FLAG-ZYG11B deletion mutants (Δ1, Δ2, Δ3, Δ4, and Δ5) were generated from FLAG-ZYG11B by PCR-based site-directed mutagenesis. GFP-ZYG11B-1–58 was generated from FLAG-ZYG11B and cloned into the pEGFP-C1 vector (6076–1, BD). All other plasmids were sourced from our laboratory.

### Cell culture and virus infection

Human Embryonic Kidney 293T, HeLa, Human Rhabdomyosarcoma RD, and African Green Monkey Kidney Vero cells were purchased from American Type Culture Collection (ATCC, Manassas, VA) and grown as monolayers in Dulbecco’s Modified Eagle Medium (DMEM, 11965-092, Gibco) supplemented with 10% heat-inactivated fetal bovine serum (FBS, ST30-3302, PAN Seratech), 1 U/mL penicillin, and 100 mg/mL streptomycin (03-031-1B, Biological Industries). Cells were maintained at 37°C in a humidified incubator with 5% CO_2_. Cells in the plate reached approximately 70% confluence; they were washed once with PBS (21–040, Corning), followed by the addition of DMEM containing the virus. The plate was incubated at 37°C for 2 h, with gentle shaking every 15 min. After infection, the medium was replaced with fresh DMEM supplemented with 10% FBS, and the cells were returned to the incubator at 37°C. EV71 strain CC063 was isolated from hand, foot, and mouth disease (HFMD) patients in 2010 described by Wang et al., and EVD68 strain US/KY/14–18953 was purchased from ATCC. CA16 strain CC024 was isolated and described by Li et al., and CA6 strain CC046 was isolated and described by Wang et al.

### Construction of stably silenced cell lines

HEK293T cells were co-transfected with the sh-ZYG11B-pLKO.1 or pLKO.1 control plasmids, along with the packaging vectors RRE, REV, and VSV-G, using Lipofectamine 2000 (11668019, Thermo), according to the manufacturer’s instructions. Lentiviral particles were harvested 48 h post-transfection, filtered, and used to infect HEK293T cells for 48 h. Following infection, stable cell lines were selected by adding puromycin (3 µg/mL, P8833, Sigma) to the culture medium.

### RNA extraction and RT-qPCR

Cells or viruses were lysed with 800 µL of Trizol (15596–026, Thermo), and 200 µL of chloroform was added to separate RNA into the aqueous phase. RNA was precipitated by combining the aqueous layer with 500 µL isopropanol and incubating overnight at −20°C. The RNA pellet was dissolved in 30 µL of DEPC-treated water. cDNA synthesis was performed using the High-Capacity cDNA Reverse Transcription Kit (Monad, MR05101M). RT-qPCR was conducted on an Mx3005P system (Agilent Technologies, Stratagene, La Jolla, CA, USA) with specific primers and SYBR Green I dye (Roche, 491314001) for detection. The cycling protocol included an initial denaturation at 95°C for 2 min, followed by 40 cycles of 95°C for 30 s (denaturation), 55°C for 30 s (annealing), and 72°C for 30 s (extension).

### Transfection

At approximately 70% confluence in the 12-well plate, plasmid DNA was mixed with PEI (23966, Polysciences) in Opti-MEM (31985–070, Thermo), incubated for 20 min, and then added to the cells. For siRNA transfection, at 20% confluence, siRNA was complexed with Lipofectamine 2000 (11668019, Thermo) in Opti-MEM, incubated for 20 min, and then applied to the cells. All siRNAs were purchased from RiboBio (Guangzhou, China).

### Western blotting

Cells were lysed in RIPA buffer (1 M Tris-7.8, 1 M NaCl, NP40, and 0.5M EDTA) and heated at 100°C for 30 min. Proteins were separated via SDS-PAGE and transferred onto PVDF membranes. Membranes were blocked with 5% non-fat milk in TBST for 1 h at room temperature, followed by overnight incubation with primary antibodies at 4°C. After 30 min washes by TBST, HRP-conjugated secondary antibodies were applied for 1 h at room temperature. Protein detection was performed using an enhanced chemiluminescence (ECL) system, and images were acquired using a digital imaging system.

### Co-immunoprecipitation (CO-IP)

Cells were collected, washed with cold PBS, and lysed in a buffer containing PBS, 1% Triton X-100, protease inhibitors (11836170001, Roche) at 4°C for 1 h on a rotator. Lysates were clarified by centrifugation at 10,000×*g* for 30 min at 4°C. The resulting supernatants were incubated with agarose beads and antibodies at 4°C for 4 h on a rotator. After incubation, samples were washed six times with cold wash buffer (20 mM Tris-HCl, 100 mM NaCl, 0.1 mM EDTA, 0.05% Tween-20; pH 7.5) and prepared for immunoblotting.

### Fluorescence resonance energy transfer (FRET)

HeLa cells were seeded in 35 mm confocal dishes and incubated overnight. The following day, cells were transfected with VP1-YFP and ZYG11B-CFP plasmids using Lipofectamine 3000 (Thermo, L3000-008). After 24 h, cells were washed with PBS and fixed in 4% paraformaldehyde at 37°C for 10 min, and fluorescence was visualized using an Olympus FV 3000 laser scanning confocal microscope.

### Protein purification

The protein of ZYG11B and vp1 was cloned into the pET-28a prokaryotic expression vector, incorporating an N-terminal GST tag or 6 × his tag. BL21 (DE3, TIANGEN, CB105) competent cells transformed constructs, and protein expression was induced in 3 L of bacterial culture using 0.5 mM IPTG (Sigma-Aldrich, 16758) when OD600 reached 0.6–0.8 overnight at 16°C with shaking at 100 rpm. The following day, bacterial cells were harvested by centrifugation at 6,000×*g* for 6 min, resuspended in 50 mL of Hepes buffer (200 mM NaCl and 20 mM Hepes, PH = 7.5), and lysed via sonication at 4°C. After centrifuging the lysate at 12,000 rpm for 30 min, the supernatant was filtered through a 0.45 µm filter and passed through Ni-NTA or GST Sepharose. The bound proteins were eluted with varying concentrations of glutathione (GSH) or imidazole. Eluted fractions were analyzed by SDS-PAGE and stained with Coomassie Brilliant Blue. Fractions containing a single target protein were selected, supplemented with 10% glycerol, and stored at −80°C for subsequent experiments.

### GST Pull-down

ZYG11B or GST protein was mixed with EV71-VP1-His protein at a 1:1 ratio and incubated with 100 µL of GST Sepharose on a rotator at 4°C for 4 h. Following centrifugation at 800 × g for 1 minute, the supernatant was discarded, and the GST Sepharose was washed 8–10 times with Hepes buffer. Proteins were eluted by adding 1 × loading buffer (0.08 M Tris, pH 6.8, 2% SDS, 10% glycerol, 0.1 M dithiothreitol, 0.2% bromophenol blue), boiled at 100°C for 10 min, and centrifuged at 12,000 rpm for 10 min. The supernatant was collected for SDS-PAGE, and protein bands were visualized by Coomassie Brilliant Blue staining.

### Microscale thermophoresis (MST)

The interaction between proteins was analyzed using the MicroScale Thermophoresis (MST) technique. For labeling, 100 µL of 200 mM VP1-His was combined with RED-tris-NTA dye and incubated in the dark for 30 min. ZYG11B-GST was serially diluted in twofold steps across 16 concentrations and incubated at room temperature for 15 min. The samples were then transferred into capillaries and analysed with the Monolith NT.115 (NanoTemper). Changes in fluorescence intensity, reflecting thermophoretic movement, were recorded. Fluorescence intensity was plotted against ligand concentration to determine the affinity constant (KD).

### Mass spectrometry analysis

293T cells were placed in a 10 cm cell culture dish and transfected with EV71-VP1-Flag the next day. The cells were harvested 48 h later and treated MG132 (10 µM) proteasome inhibitor 16 h before harvesting. The cell lysate was incubated with Flag beads at 4°C overnight, competed with Flag peptide at 4 °C for 4 h, and separated the proteins by SDS-PAGE. After separation by SDS-PAGE and Coomassie Brilliant Blue staining, the samples were sent to the core facility at the First Hospital of Jilin University for mass spectrometry analysis.

### Statistical analysis

The statistical analysis used has been described in detail in the legend. All data are presented as mean ± standard deviation (SD). Statistical comparisons are conducted using Student’s *t*-test, one-way ANOVA, or repeated-measures ANOVA. The differences are statistically significant: *P < 0.05, **P < 0.01, ***P < 0.001; ns represents meaninglessness.

## Data Availability

The mass spectrometry proteomics data have been deposited in the ProteomeXchange Consortium (https://proteomecentral.proteomexchange.org) via the iProX partner, with the dataset identifier PXD059489. All other data supporting the results of this study can be obtained in the paper and its supplemental files.

## SUPPLEMENTAL MATERIAL

Supplemental material jvi.00030-25-s0001.pdf Figures S1 to S7; Tables S1 and S2.

ASM does not own the copyrights to Supplemental Material that may be linked to, or accessed through, an article. The authors have granted ASM a non-exclusive, world-wide license to publish the Supplemental Material files. Please contact the corresponding author directly for reuse.
