# Supplementary material for: ZYG11B suppresses multiple enteroviruses by triggering viral VP1 degradation
Source: J Virol. 2025 Mar 26;99(4):e00030-25. doi: 10.1128/jvi.00030-25 (PMC11998487; doi:10.1128/jvi.00030-25)
Supplement: Supplemental material — Figures S1 to S7; Tables S1 and S2. [file jvi.00030-25-s0001.pdf]

ZYG11B suppresses multiple enteroviruses by triggering viral VP1 degradation

Li Tian,[2#] Zhizhong Mi,[2#] Weijing Yang,[2] Jing Chen,[2] Xiulong Wei,[2] Wenyan Zhang [1, 2*] and Zhaolong Li [1, 2*]

[1]Department of Infectious Diseases, Infectious Diseases and Pathogen Biology Center, Key Laboratory of Organ Regeneration and Transplantation of The Ministry of Education, The First Hospital of Jilin University, Changchun, Jilin, China;

[2]Institute of Virology and AIDS Research, the First Hospital of Jilin University, Changchun, Jilin, China;

Running Head: ZYG11B has broad spectrum anti-enterovirus

*Address correspondence to Zhaolong Li, lizhaolong@jlu.edu.cn.

*Address correspondence to Wenyan Zhang, zhangwenyan@jlu.edu.cn.

#These authors contributed equally to this article. Author order was determined by drawing lots.

This PDF file includes:

Appendix Fig S1-S7

Appendix Table S1-S2

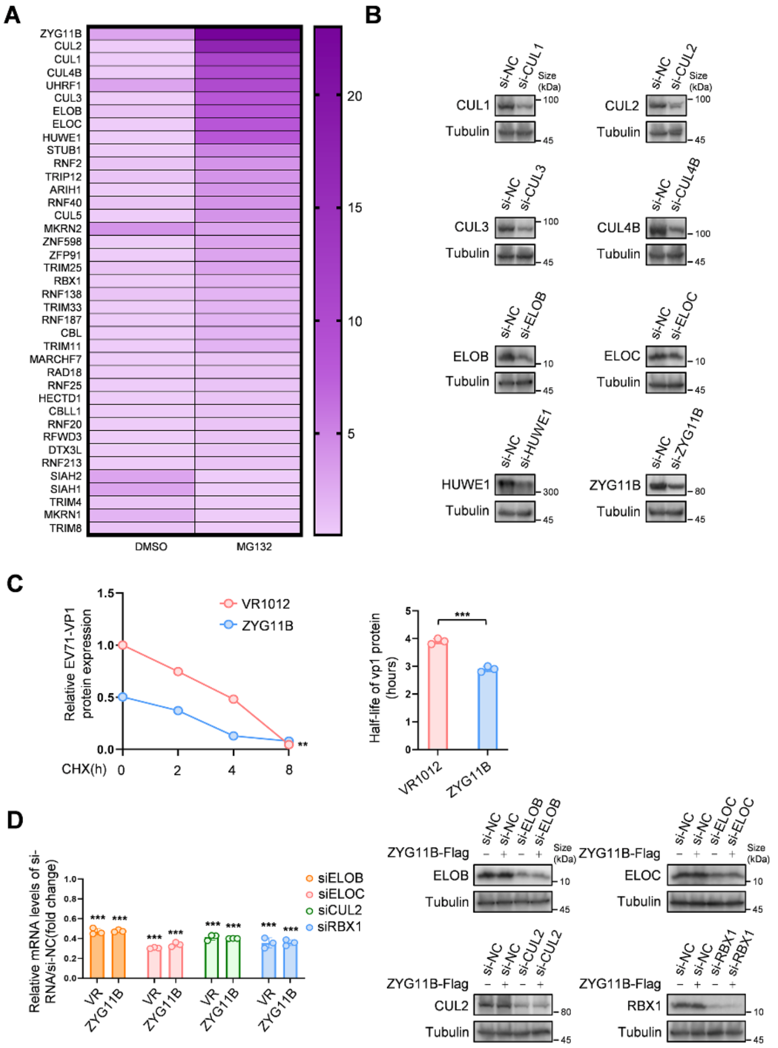

Fig S1 ZYG11B interacts with and degrades EV71 VP1 via the proteasomal pathway. (**A**) Heatmap analysis of the mass spectrometry data identifies proteins interacting with EV71-VP1 and associated with the proteasomal pathway. Transfect EV71-VP1-HA and add MG132 or DMSO separately for treatment. EV71-VP1 was enriched using protein-G with HA antibody, and the proteins were separated by SDS-PAGE and analyzed by mass spectrometry (MS). Screen proteins that interact with EV71-VP1 of MG132 treatment group was found to be greater than that enriched in DMSO treatment group. Analyze the proteins or complexes with E3 ubiquitin ligase function in the EV71-VP1 interacting protein and draw a heatmap. (**B**) Detect the knockdown efficiency of siRNA in Fig. 1E by IB. (**C**) Transfect ZYG11B, the half-life of EV71-VP1 was significantly reduced. Quantify the G graph of Figure 1 using image J and calculate its half-life. (**D**) The knockdown efficiency of each factor in Fig. 1H was assessed by RT-qPCR, represented as the ratio to the corresponding NC group factor levels, and by IB to detect the corresponding protein levels. The data is representative of three independent experiments, expressed as mean ± standard deviation (n=3). Student t-test (unpaired, two-way) was used for comparison between two independent groups: **p < 0.01; ***p < 0.001.

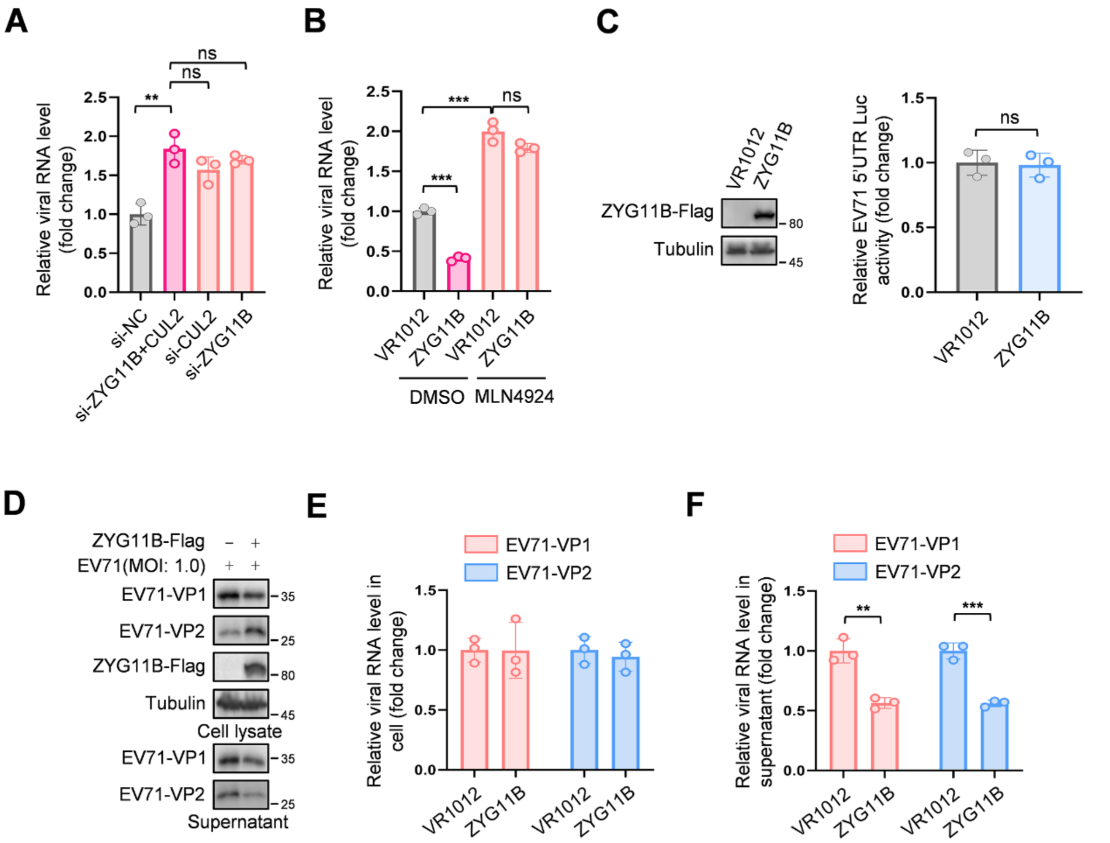

Fig.S2 ZYG11B's antiviral effect does not affect viral replication and translation. (**A**) The viral RNA levels in the supernatant of panels G and H of Figure 2. (**B**) The viral RNA levels in the supernatant of panels I and J of Figure 2. (**C**) ZYG11B does not affect the activity of EV71's 5' UTR. The 5' UTR of EV71 was cloned into the pIRIGF vector (Addgene, catalog number 101139). The bicistronic 5' UTR-pIRIGF expression vector was co-transfected with ZYG11B or VR1012. After 48 hours, cells were harvested, and luciferase activity was measured using a Fluoroskan Ascent FL (Thermo Fisher) and Dual-Luciferase Reporter Assay System (Promega). (**D-F**) ZYG11B does not affect viral RNA replication. ZYG11B or VR1012 was transfected into cells, and after 24 hours, the cells were infected with the virus at an MOI of 1.0. After 2 hours, the medium was replaced, and cells and supernatants were harvested after 8 hours. The levels of viral structural proteins VP1 and VP2 in cells and supernatants were detected by IB and RT-qPCR. The data is representative of three independent experiments, expressed as mean ± standard deviation (n=3). Student t-test (unpaired, two-way) was used for comparison between two independent groups, and two-way ANOVA was used for comparison between multiple groups: ns; $^{**}p < 0.01$; $^{***}p < 0.001$.

A

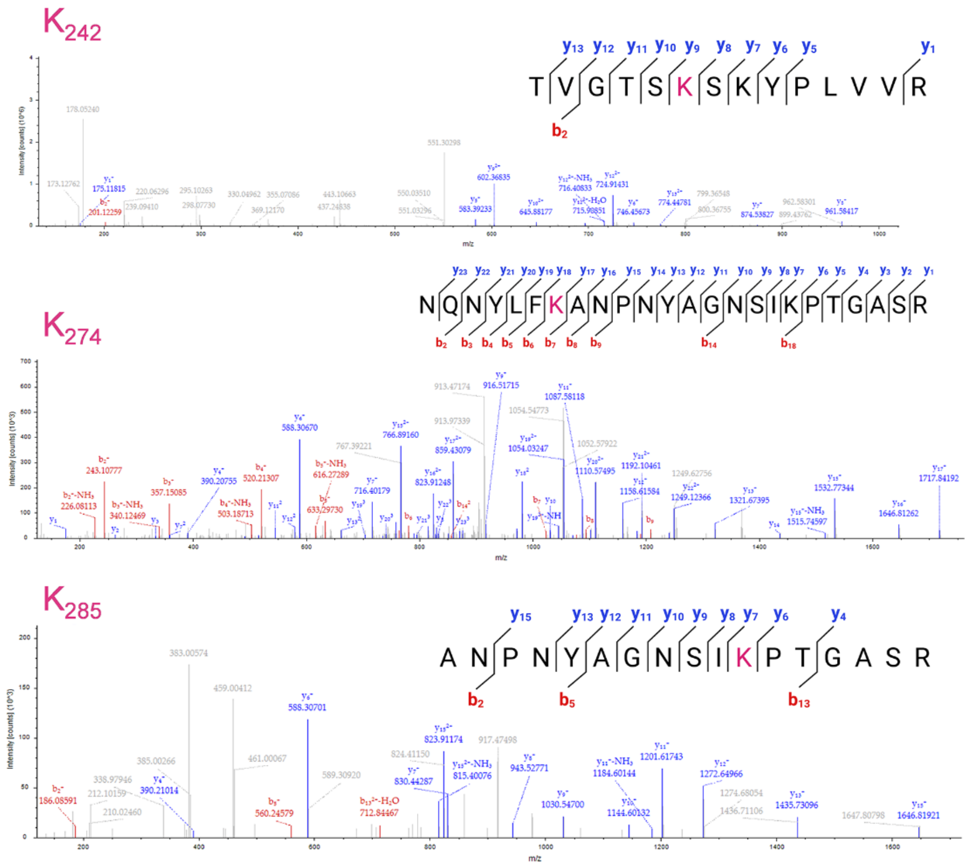

Fig S3 Raw data of mass spectrometry analysis for EV71-VP1 ubiquitination sites.

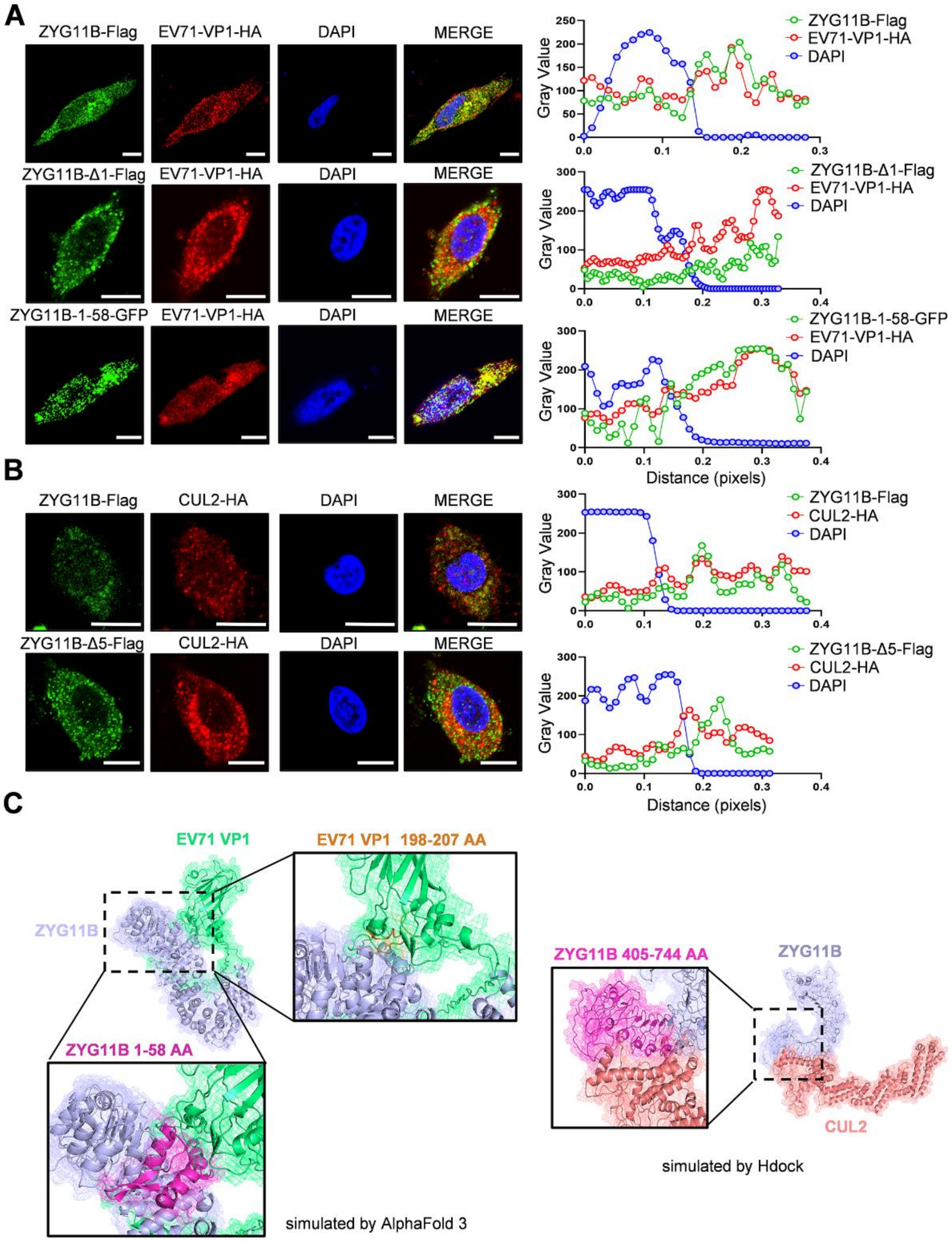

Fig S4 Proving the interaction region between ZYG11B and EV71-VP1 or CUL2 through immune co-localization and structural docking. (**A and B**) In HELA cells, ZYG11B or its mutants were co-transfected with EV71-VP1 or CUL2. After 36 hours, MG132 (10 μM) was added and the cells were treated for 12 hours. After drug treatment, the protein was labeled overnight with HA antibody and Flag antibody. Then, it was fluorescently labeled with 488 (green: ZYG11B or its mutants) and 568 (red: CUL2 or EV71-VP1) fluorescent secondary antibody. Cell nuclei were stained using DAPI (blue). Representative images were shown. Scale bars, 10 μm. The ratio of colocalization was quantified by measuring the fluorescence intensities using Image J. (**C**) Docking predictions of ZYG11B with CUL2 and ZYG11B with VP1 were performed using the online tools HDOCK and AlphaFold3.

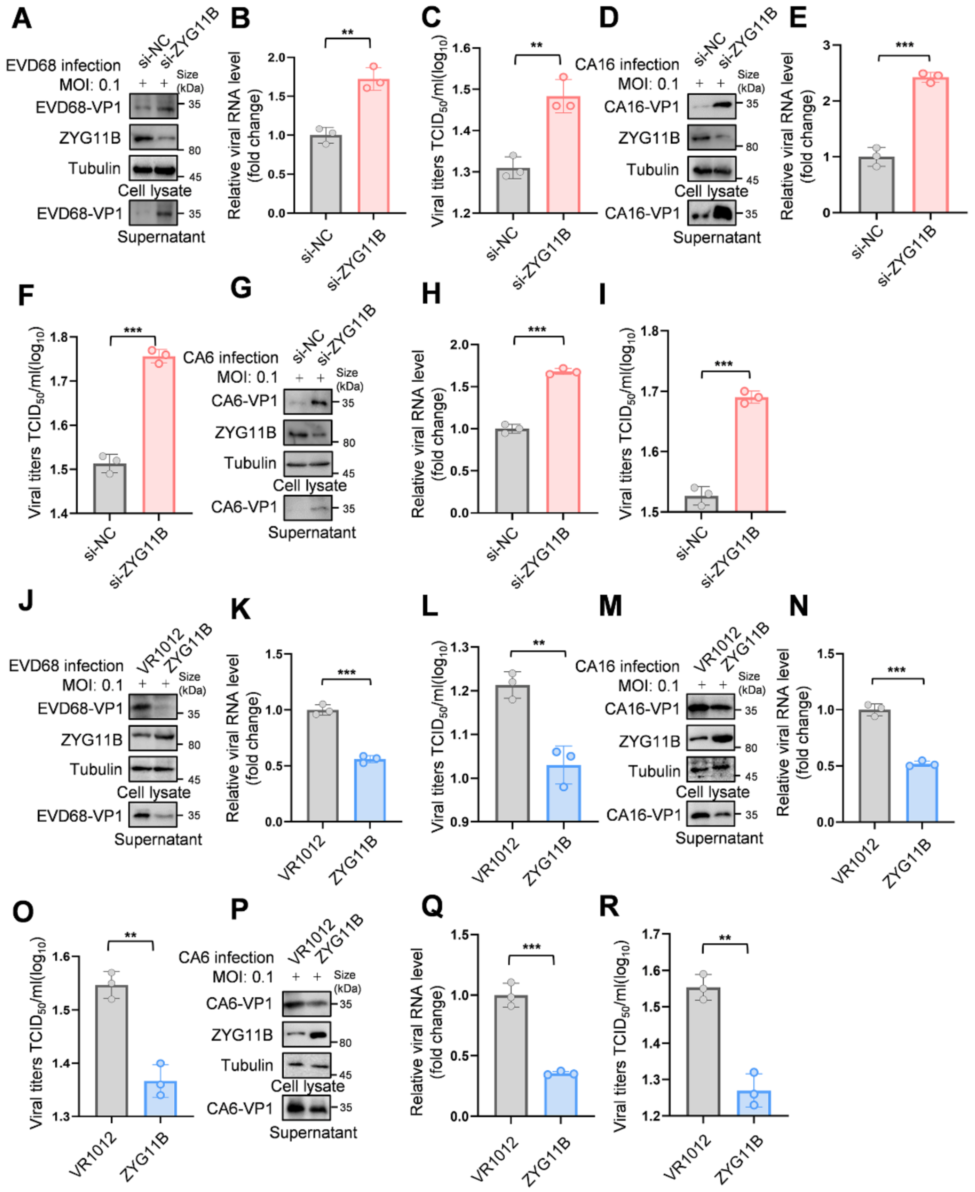

Fig S5 ZYG11B has a broad spectrum of anti-enterovirus ability. (**A-I**) Knockdown of ZYG11B factor levels enhanced viral replication. 293T cells were transfected with siRNA targeting ZYG11B or NC (a random sequence without targeting), infected with EVD68 (**A-C**), CA16 (**D-F**) or CA6 (**G-I**) (MOI:0.1) 24 h later. After 8 hours, replace the supernatant with clean culture medium and incubate for 40 hours. The cells as well as the supernatant were harvested to be measured for viral levels. EV71-VP1 protein levels in cells and supernatant were determined by IB. The viral RNA level in the supernatant was detected by RT-qPCR, and the viral titer in the supernatant was detected. (**J-R**) Overexpression of ZYG11B was able to resist the virus. Cells were transfected with VR1012 or ZYG11B-Flag expression plasmids and infected with EVD68 (**J-L**), CA16 (**M-O**), or CA6 (**P-R**) (MOI:0.1) 24 h later. Then changed culture medium 8 h later. After 48 hours, cells and supernatant were harvested and other procedures were performed as above. The data is representative of three independent experiments, expressed as mean ± standard deviation (n=3). Student t-test (unpaired, two-way) was used for comparison between two independent groups, **p < 0.01; ***p < 0.001.

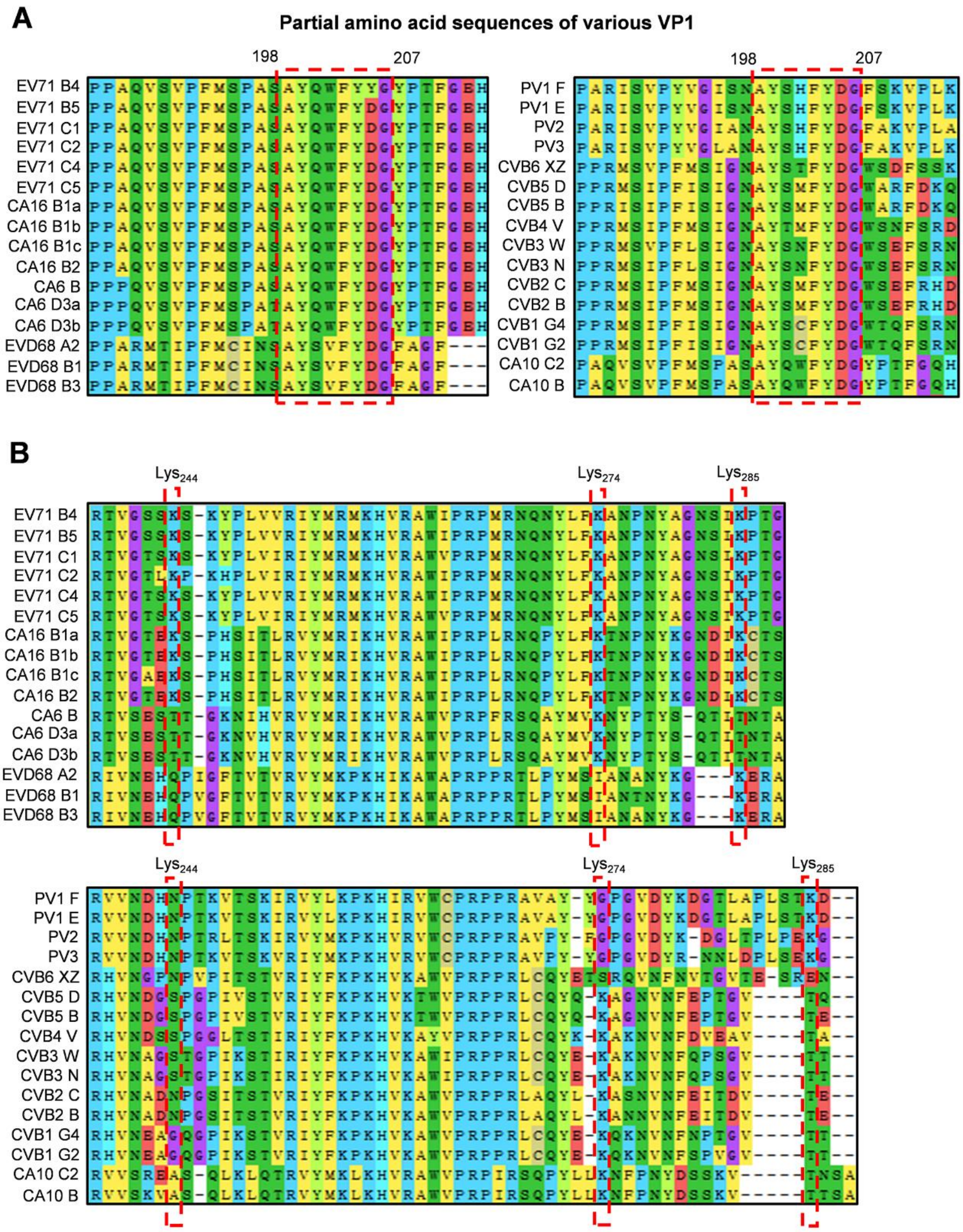

Fig S6 The interaction region with ZYG11B in VP1 sequences of epidemic enterovirus strains is conserved, and its ubiquitination site is also relatively conserved. (**A and B**) The VP1 sequences of epidemic EV71, EVD68, CA6, CA16, CVB, PV, CA10 strains were obtained from NCBI, and the sequences were compared by Molecular Evolutionary Genetics Analysis software.

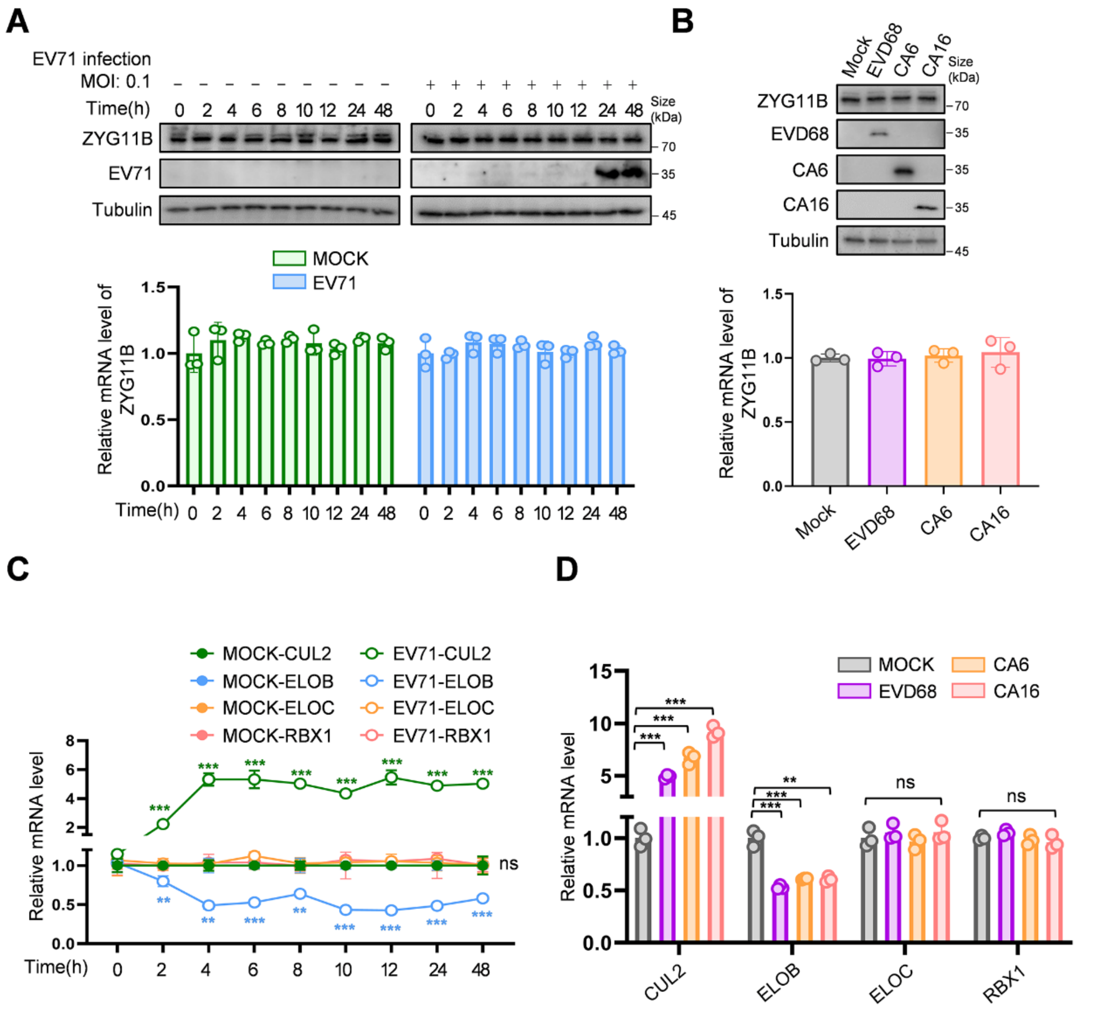

Fig S7 Enterovirus infection did not affect ZYG11B expression but down-regulated ELOB RNA levels. (**A**) The level of ZYG11B RNA or protein were not adjusted by EV71 infection. The RD cells were infected with EV71 virus (MOI: 0.1). Respectively at different time points (0 h, 2 h, 4 h, 6 h, 8 h, 10 h, 12 h, 24 h, 48 h) harvest cells. The sample average into 2 portions, one of which was used for IB, and the protein level was measured by EV71-VP1 antibody or ZYG11B antibody. The other was used for RNA extraction and RNA levels were measured by RT-qPCR as well as the indicated primers. (**B**) Infection with other enteroviruses did not affect ZYG11B protein level or RNA level. RD cells were separately infected with CA6, CA16 or EVD68 (MOI: 0.1), and cells were harvested at 24 h. The other procedures were the same as in Figure S7A. (**C**) The level of ELOB RNA in CUL2 complex was down-regulated after EV71 infection. RD cells were infected with EV71 (MOI: 0.1), and cells were harvested at different time points (0 h, 2 h, 4 h, 6 h, 8 h, 10 h, 12 h, 24 h, 48 h) to measure the RNA levels of different factors by RT-qPCR. (**D**) Infection with other enteroviruses down-regulated ELOB RNA levels. RD cells were infected with CA6, CA16 or EVD68 (MOI: 0.1), and cells were harvested at 24h to measure the RNA levels of different factors by RT-qPCR. The data is representative of three independent experiments, expressed as mean ± standard deviation (n=3). Student t-test (unpaired, two-way) was used for comparison between two independent groups, and two-way ANOVA was used for comparison between multiple groups: ns; $^{**}p < 0.01$; $^{***}p < 0.001$.

Table S1 Reagents and Tools Table

Reagent/Resource	Reference or Source	Identifier or Catalog Number
**Experimental Models**		
HEK293T	ATCC	CRL-11268
HeLa	ATCC	CCL-2
Human Rhabdomyosarcoma RD	ATCC	CCL-136
African Green Monkey Kidney Vero cells	ATCC	CCL-81
**Recombinant DNA**		
pCDNA3.1-YFP	Addgene	13033
pLKO.1-puro	Addgene	8453
pIRIGF	Addgene	101139
pECFP-C1	Addgene	Jan-76
VR1012	This study	N/A
pLKO.1-sh-ZYG11B	This study	N/A
VR1012-EV71-VP1-HA	This study	N/A
VR1012-CUL2-Flag	This study	N/A
VR1012-ELOB-Flag	This study	N/A
VR1012-ELOC-Myc	This study	N/A
VR1012-RBX1-Flag	This study	N/A
VR1012-EV71 infectious clone	This study	N/A
VR1012-CA6-VP1-HA	This study	N/A
VR1012-CA16-VP1-HA	This study	N/A
VR1012-EVD68-VP1-HA	This study	N/A
VR1012-ZYG11B-Flag	This study	N/A
pIRIGF-EV71-5'UTR	This study	N/A
**Antibodies**		
Mouse monoclonal anti-HA	Biolegend	Cat#901514; RRID: AB_2565336
Rabbit monoclonal anti-HA	invitrogen	Cat#715500; RRID: AB_2533988
Mouse monoclonal anti-Flag	Sigma	Cat#F1804; RRID: AB_262044
Rabbit monoclonal anti-Flag	Proteintech	Cat#20543-1-AP; RRID: AB_11232216
beta Tubulin Polyclonal Antibody	Proteintech	Cat#10068-1-AP; RRID: AB_2303998
EV71 VP1 antibody	GENETEX	Cat#GTX132339; RRID: AB_2886617
EV71 VP2 antibody	GENETEX	Cat#GTX132340; RRID: AB_2886618
EVD68 VP1 antibody	GENETEX	Cat#GTX132313; RRID: AB_2886609
CA6 VP1 antibody	GENETEX	Cat#GTX132346; RRID: AB_2886622
Mouse Anti-GST Tag Monoclonal Antibody	BBI	Cat#D190101; RRID: AB_2940945
Goat anti-Mouse IgG (H+L) Cross-Adsorbed Secondary Antibody, Alexa Fluor 488	Thermo	Cat#A-11001; RRID: AB_2534069
Goat anti-Rabbit IgG (H+L) Cross-Adsorbed Secondary Antibody, Alexa Fluor 568	Thermo	Cat#A-11011; RRID: AB_143157
HRP goat-anti Mouse	Jackson	Cat#115-035-062; RRID: AB_2338504

HRP goat anti Rabbit	Jackson	Cat#111-035-045; RRID: AB_2337938
Anti-6×His Mouse antibody	Abcam	Cat#ab18184; RRID: AB_444306
GFP-tag (ME11) Mouse Monoclonal Antibody	RayBiotech	Cat#RM1008; RRID: AB_2756458
CUL1 Monoclonal antibody	Proteintech	Cat# 66978-1-Ig; RRID: AB_2886486
CUL2 Monoclonal antibody	Proteintech	Cat# 67175-1-Ig; RRID: AB_2886487
CUL3 Polyclonal antibody	Proteintech	Cat# 11107-1-AP; RRID: AB_10863339
TCEB2/Elongin-B Polyclonal antibody	Proteintech	Cat# 10779-1-AP; RRID: AB_10863174
TCEB1 Monoclonal antibody	Proteintech	Cat# 68164-1-Ig; RRID: AB_2886485
RBX1 Polyclonal antibody	Proteintech	Cat# 14895-1-AP; RRID: AB_2886484
HUWE1 Polyclonal antibody	Proteintech	Cat# 19430-1-AP; RRID: AB_2886489
CUL4B Polyclonal antibody	Proteintech	Cat# 12916-1-AP; RRID: AB_2886488
ZER1 Polyclonal antibody	Proteintech	Cat# 16647-1-AP; RRID: AB_2886483
**Chemicals, Enzyme and other reagents**		
DAPI	Thermo	Cat#D1306; RRID: AB_2629482
Dulbecco’s modified Eagle’s medium	Thermo	11995065
Fetal Bovine Serum	PAN Seratech	ST30-3302
Opti-MEM	Thermo	31985-070
Penicillin-streptomycin Solution	Biological Industries	03-031-1B
Polyethylenimine(PEI)	Polysciences	23966
Lipofectamine 2000 Reagent	Thermo	11668019
Lipofectamine 3000 Reagent	Thermo	L3000-008
DMSO	Sigma	D8418
MG132	Selleck	S2619
MLN4924	Selleck	S7109
Protease inhibitor cocktail	Roche	11836170001
Bortezomib	Selleck	S1013
Protein G-Agarose	Roche	11243233001
Trizol Reagent	Thermo	15596-026
MonScript RTIII All-in-One Mix with dsDNase	Monad	MR05101M
MonAmp ChemoHS qPCR Mix	Monad	MQ00401S
Meilunbio fgsuper sensitive ECL luminescence reagent	Meilunbio	MA0186-1
Sodium Chloride	Sigma-Aldrich	7647-14-5
PBS	Corning	21-040
SDS	Sigma-Aldrich	151-21-3
IPTG	Sigma-Aldrich	16758
HEPES	Gibco	15630-080
puromycin	Sigma	P8833
**Software**		
PDB	www.rscb.org	
NCBI	www.ncbi.nlm.nih.gov	

GraphPad Prism 9	GraphPad Software	https://www.graphpad.com/
Image J	NIH	https://imagej.nih.gov/ij/docs/intro.html/
Hdock	HDOCK server	http://hdock.phys.hust.edu.cn/
pymol	Schrödinger	www.pymol.org/

Table S2 PCR primers

Primers	Sequence (5'-3')
ZYG11B△1-F：	CAAGGACGACGATGACAAG
ZYG11B△1-R：	GCGCCTCCTCCATTGCTGCG
ZYG11B△2-F：	GGTGGCTGATCGACTGGCTGAAGTTG
ZYG11B△2-R：	GTCGATCAGCCACCTCCTGTGGG
ZYG11B△3-F：	GTTCTCTTTTACAATGAACTTCGCTTACTAGAAC
ZYG11B△3-R：	GTTCATTGTAAAAGAGAACATTCGTGAT
ZYG11B△4-F：	TCTCTGGATGTTTCTGATGTGACCCATTTG
ZYG11B△4-R：	CAGAAACATCCAGAGAAACAAGGTT
ZYG11B△5-F：	CCGACTCCTGGCTTAGGATGTGACCCATTTG
ZYG11B△5-R：	CAGCCAGGAGTCGGACAGGCATC
ZYG11B-RT-MUS-F：	CTGAATGATGGAACTGTGGG
ZYG11B-RT-MUS-R:	CTGAAGATTTTGCTGGATCC
ZYG11B-sh-F:	CCGGGCTTTCTGCCACCACAAGTTACTCGAGTAACTTGTGGTGGCAGAAAGCTTTTTG
ZYG11B-sh-R：	AATTCAAAAAGCTTTCTGCCACCACAAGTTACTCGAGTAACTTGTGGTGGCAGAAAGC
ZYG11B-RT-F：	GCTTGTCATGCAGTGGCTTTGC
ZYG11B-RT-R：	GCTCAGTACCAAGCTGTGCAGT
CUL1-RT-F：	CAATGACGCTGGCTTTGTGGCT
CUL1-RT-R：	CAAGGAGTCACAGTATCGAGCC
CUL2-RT-F：	GTCTTACTCCGTGCTGTGTCCA
CUL2-RT-R：	CTGACTCCACAAATAGTGTTGGC
CUL3-RT-F：	TCGACAGCTCACACTCCAGCAT
CUL3-RT-R：	GTGCTTCCGTGTATTAGAGCCAG
CUL4b-RT-F：	GAAGCTACAGATGAAGAACTTGAG
CUL4b-RT-R：	GCACTCTTTCCGACTAACAGGC
ELOB-RT-F：	GCTTCACCAGTCAAACAGCACG
ELOB-RT-R：	GTTCATTGGCACTGCTTCCCGA
ELOC-RT-F：	AAGAGAACATGCATTAACATCAGGC
ELOC-RT-R：	TGCATACTTTCGATAGCACATGTG
HUAWE1-RT-F：	CGGCTTTTCCTGAAGAAGGGAC
HUAWE1-RT-R：	GTTTCCAAAGGCTTCAGAGCAGC
RBX1-RT-F：	ACTGTGCCATCTGCAGGAACCA
RBX1-RT-R：	ACCTGTCGTGTTTTGAGCCAGC
EV71-VP1-RT-F:	AGCACCCACAGGCCAGAACACAC
EV71-VP1-RT-R:	ATCCCGCCCTACTGAAGAAACTA
EV71-VP2-RT-F:	ACCATCACCACGCAAGAAG
EV71-VP2-RT-R:	TGCATTTTGCCCAAAAACCC
